# Large Proteins Have a Great Tendency to Aggregate but a Low Propensity to Form Amyloid Fibrils

**DOI:** 10.1371/journal.pone.0016075

**Published:** 2011-01-13

**Authors:** Hassan Ramshini, Claudia Parrini, Annalisa Relini, Mariagioia Zampagni, Benedetta Mannini, Alessandra Pesce, Ali Akbar Saboury, Mohsen Nemat-Gorgani, Fabrizio Chiti

**Affiliations:** 1 Dipartimento di Scienze Biochimiche, Università di Firenze, Florence, Italy; 2 Institute of Biochemistry and Biophysics, University of Tehran, Tehran, Iran; 3 Dipartimento di Fisica, Università di Genova, Genoa, Italy; University of South Florida College of Medicine, United States of America

## Abstract

The assembly of soluble proteins into ordered fibrillar aggregates with cross-β structure is an essential event of many human diseases. The polypeptides undergoing aggregation are generally small in size. To explore if the small size is a primary determinant for the formation of amyloids under pathological conditions we have created two databases of proteins, forming amyloid-related and non-amyloid deposits in human diseases, respectively. The size distributions of the two protein populations are well separated, with the systems forming non-amyloid deposits appearing significantly larger. We have then investigated the propensity of the 486-residue hexokinase-B from *Saccharomyces cerevisiae* (YHKB) to form amyloid-like fibrils *in vitro*. This size is intermediate between the size distributions of amyloid and non-amyloid forming proteins. Aggregation was induced under conditions known to be most effective for amyloid formation by normally globular proteins: (i) low pH with salts, (ii) pH 5.5 with trifluoroethanol. In both situations YHKB aggregated very rapidly into species with significant β-sheet structure, as detected using circular dichroism and X-ray diffraction, but a weak Thioflavin T and Congo red binding. Moreover, atomic force microscopy indicated a morphology distinct from typical amyloid fibrils. Both types of aggregates were cytotoxic to human neuroblastoma cells, as indicated by the MTT assay. This analysis indicates that large proteins have a high tendency to form toxic aggregates, but low propensity to form regular amyloid *in vivo* and that such a behavior is intrinsically determined by the size of the protein, as suggested by the *in vitro* analysis of our sample protein.

## Introduction

A large family of human pathologies is associated with the conversion of peptides and proteins from their soluble functional forms into well-defined fibrillar aggregates, often called amyloid fibrils when they accumulate in the extracellular space [1]. Such diseases include neurodegenerative disorders, such as Alzheimer's disease and spongiform encephalopathies, non neuropathic localized amyloidoses, such as type II diabetes and atrial amyloidosis and non neuropathic systemic amyloidoses, like light-chain amyloidosis and dialysis-related amyloidosis [1]. Images acquired by transmission electron microscopy show that amyloid fibrils are long, rigid, unbranched and usually consist of a number (typically 2–6) of protofilaments, each about 2–5 nm in diameter [2]. These protofilaments twist together to form rope-like fibrils that are typically 7–13 nm wide [2], [3] or associate laterally to form long ribbons that are 2–5 nm thick and up to 30 nm wide [4], [5]. The fibrils have the ability to bind specific dyes such as thioflavin T (ThT) and Congo red (CR) [6] and are characterized by an extended cross-β structure, as revealed by X-ray fiber diffraction [3].

The peptides and proteins that form extracellular amyloid fibrils, or intracellular inclusions with recognized related morphological and structural characteristics, are generally small in size, often shorter than 250 residues [1]. Even proteins that have been converted into amyloid-like fibrils *in vitro* and have no link to human diseases are generally small, typically shorter than 150 residues [7], [8]. The small percentage of large proteins recognized to form amyloid or amyloid-like fibrils is disproportionate to the fraction of such proteins in the human proteome, as more than 50% of natural human proteins are longer than 250 residues. The question thus arises as to why diseases associated with amyloid or amyloid-like deposits do not generally arise from large proteins.

To address this issue we have carried out an extensive search in the literature of all proteins recognized to form deposits distinct from amyloid under pathological conditions and have compared the sizes of such proteins with those known to form amyloid deposits in disease. We will show that the size distributions of proteins forming amyloid and non-amyloid deposits in pathology are well separated and that proteins associated with non-amyloid deposits are remarkably longer. We have then investigated the aggregation process *in vitro* of a fairly large model protein, namely the 486-residue (55 kDa) protein hexokinase-B from the yeast *Saccharomyces cerevisiae* (YHKB). The size of this protein falls within the region of overlap of the size distributions of amyloid and non-amyloid forming proteins. In particular, we have determined the type of protein aggregates formed by such protein under two sets of conditions, both shown to be among the most effective in promoting amyloid fibril formation *in vitro* [9]–[23]. Using different biophysical techniques we will show that both conditions promote the very rapid formation of aggregates, characterized by significant β-sheet structure but only weak ability to bind ThT and CR and a morphology different from that of regular amyloid fibrils. Importantly, however, we will also show that both types of aggregates appear to be toxic to cultured cells.

The results indicate that large proteins have a great tendency to aggregate into deleterious species but a poor propensity to form real amyloid fibrils and suggest that the difference in behavior between proteins forming amyloid structures and proteins producing non-amyloid deposits in pathology is primarily and intrinsically determined by the length of the polypeptide chain undergoing aggregation.

## Materials and Methods

### Materials

8-anilinonaphthalene-1-sulfonate (ANS), trifluoroethanol (TFE), trifluoroacetic acid (TFA), ThT, CR and α-cyclodextrin were purchased from Sigma-Aldrich (St. Louis, MO, USA). YHKB was also obtained from Sigma-Aldrich as a crystalline suspension in ammonium sulfate (code H6380). After re-suspension of the enzyme in the buffer used in the reaction medium, it was dialyzed at 4°C for 24 hours and then filtered with a 20-nm syringe filter (Whatman, Maidstone, UK). Protein concentration was determined by absorbance measurements at 280 nm, using an extinction coefficient (ε^1%^) of 9.47 [24]. Far-UV circular dichroism (CD) analysis confirmed the native structure of the protein. Dynamic light scattering measurements carried out with a Malvern Zetasizer Nano S instrument (Malvern, Worcestershire, UK) confirmed the absence of large aggregates in the sample.

### Far-UV CD

Far-UV CD spectra of native and acid-unfolded YHKB, both in their monomeric forms, were acquired in 50 mM phosphate buffer, pH 7.0 and in 20 mM TFA, pH 1.7, respectively. For the pH titration spectra were acquired in 20 mM glycine-HCl, 20 mM acetate; 20 mM N-(2-Hydroxyethyl)piperazine-N'-(2-ethanesulfonic acid) (HEPES); 20 mM Tris. For the TFE titration spectra were acquired in 50 mM acetate buffer, pH 5.5, and various TFE concentrations ranging from 0 to 45% (v/v), with and without 1 mM α-cyclodextrin. Far-UV CD spectra during aggregation were acquired in 20 mM TFA, 360 mM NaCl, pH 1.7 (first explored conditions) and in 50 mM acetate buffer, pH 5.5, 30% (v/v) TFE (second explored conditions) at various time points. Spectra were also recorded in 20 mM TFA, pH 1.7 at various NaCl concentrations ranging from 30 to 810 mM. In all these experiments YHKB was diluted to a final concentration of 0.1 mg/ml and the spectra were recorded at 25°C using a 1-mm path-length cell and a Jasco J-810 spectropolarimeter (Tokyo, Japan) equipped with a thermostated cell holder connected to a Thermo Haake C25P water bath (Karlsruhe, Germany).

### Intrinsic fluorescence spectroscopy

pH titration and TFE titration were also followed by recording fluorescence spectra of YHKB, at a concentration of 0.025 mg/ml, in the conditions described above for far-UV CD. The spectra were recorded at 25°C using a Perkin-Elmer LS 55 spectrofluorimeter (Wellesley, MA) equipped with a thermostated cell holder attached to a Haake F8 water bath (Karsruhe, Germany). A 2×10 mm path-length quartz cell and an excitation wavelength of 280 nm were used.

### ThT assay

YHKB was incubated at 25°C, at a protein concentration of 0.1 mg/ml, in (a) 20 mM TFA, pH 1.7, 25°C and NaCl at concentrations ranging from 30 to 810 mM and (b) 50 mM acetate buffer, pH 5.5, 30% (v/v) TFE, 25°C. At different time points, 60 µl aliquots of each sample were mixed with 440 µl of a solution containing 25 µM ThT, 25 mM sodium phosphate buffer, pH 6.0, 25°C. The fluorescence emission spectra were recorded at 25°C using an excitation of 440 nm and the instrument described above. All spectra are reported after subtracting the spectra recorded for ThT in the absence of protein from those recorded in its presence.

### CR assay

Samples were prepared similarly to the ThT experiments described above. At different time intervals, 60 µl aliquots of each sample were mixed with 440 µl of a solution containing 20 µM CR, 5 mM sodium phosphate buffer, 150 mM NaCl, pH 7.4, 25°C. Optical absorption spectra were acquired from 400 to 700 nm, after a two to three minute equilibration at 25°C, using a 5 mm path-length cell and a Jasco V-630 UV-visible spectrophotometer (Tokyo, Japan). In each case, the difference spectrum was obtained by subtracting the spectra acquired for the protein alone and for CR alone from the spectrum recorded for the protein in the presence of CR.

### Tapping-mode atomic force microscopy (TM-AFM)

Aliquots of YHKB at a concentration of 0.1 mg/ml in 30% (v/v) TFE, 50 mM acetate buffer, pH 5.5, were withdrawn at different aggregation times and diluted 5 times with milliQ water. 20 µl of the diluted samples were deposited on a freshly cleaved mica substrate and dried under vacuum. 20 µl aliquots of YHKB at a concentration of 0.1 mg/ml in 360 mM NaCl, 20 mM TFA, pH 1.7 were withdrawn at different aggregation times, deposited for 5 minutes on a freshly cleaved mica substrate, washed with milliQ water and dried under vacuum. TM-AFM images were acquired in air using a Dimension 3000 microscope (Digital Instruments–Veeco, Santa Barbara, CA), equipped with a ‘G’ scanning head (maximum scan size 100 µm) and driven by a Nanoscope IIIa controller. Single beam uncoated silicon cantilevers (type OMCL-AC160TS, Olympus, Japan) were used. The drive frequency was between 341 and 342 kHz. The scan rate was between 0.6 and 1.0 Hz. Aggregate sizes were measured from the height in cross section of the topographic AFM images. To take into account the reduction in size with respect to fully hydrated conditions caused by the drying procedure, the aggregate heights reported in the Results were obtained multiplying the measured heights by a shrinking factor of 2.2, evaluated by comparing the heights of a globular protein under liquid and in air after drying under vacuum.

### X-ray fiber diffraction

YHKB aggregates grown for 2 hours at a concentration of 0.1 mg/ml in a solution containing 30% (v/v) TFE, 50 mM acetate buffer, pH 5.5, were centrifuged (3500 rpm, 21°C) for 10 minutes. The resulting pellet was collected and inserted in a 0.7 mm capillary with an open end. The capillary was then inserted into an expanded polystyrene support to avoid its rupture and gently centrifuged. After removal of the supernatant the pellet was exposed to air for about 1 week. The capillary was exposed to X-rays at room temperature using synchrotron radiation at beamline ID14-4 (ESRF, Grenoble). Analysis of fibre diffraction images and estimate of spacings was achieved using the program Mosflm [25].

### 3-(4,5-dimethylthiazol-2-yl)-2,5-diphenyltetrazolium bromide (MTT) reduction test

Human neuroblastoma cells (SH-SY5Y) (A.T.C.C., Manassas, VA) were cultured in Dulbecco's Modified Eagle's Medium (DMEM) F-12 Ham with 25 mM HEPES and NaHCO_3_ (1∶1) supplemented with 10% fetal bovine serum (Sigma-Aldrich), 1.0 mM glutamine and antibiotics. Cells were maintained in a 5.0% CO_2_ humidified atmosphere at 37°C and grown until 80% confluence for a maximum of 20 passages. YHKB samples were prepared similarly to the ThT experiments described above. At various time intervals aliquots were centrifuged, dried under N_2_ to remove TFE or TFA when necessary, dissolved in DMEM without phenol red, and immediately added to SH-SY5Y cell culture media for 24 h at a final YHKB concentration of 2 µM. Aggregate cytotoxicity was assessed in 96-well plates by the MTT assay (Sigma, Milan, Italy), as previously reported [26]. Briefly, after the exposure to 2 µM YHKB aggregates for 24 h at 37°C, the cell cultures were incubated with 0.5 mg/ml MTT solution at 37°C for 4 h and with cell lysis buffer (20% SDS, 50% N,N-dimethylformamide, pH 4.7) for 3 hours. Absorbance values of blue formazan were determined at 590 nm. Cell viability was expressed as percent of MTT reduction in treated cells as compared to cognate untreated cells.

## Results

### Proteins forming amyloid deposits are smaller than those forming non-amyloid deposits

We have collected two databases of peptides or proteins reported to form proteinaceous deposits in pathology. The first database includes 25 peptides and proteins reported to form either extracellular amyloid fibrils or intracellular inclusions with morphological and structural amyloid-like characteristics in human pathology. The second includes 9 systems found to form deposits with characteristics distinct from amyloid in disease. Assignment to either group was based on reported evidence on the amyloid or non-amyloid nature of the deposits, as detected using electron microscopy and/or CR staining. Only proteins for which such evidence has been reported have been included in the two databases. Proteins shown to form amyloid-like fibrils only in vitro in the absence of a characterization of their amyloid morphology in vivo have been excluded from the analysis (these include α-synuclein, superoxide dismutase, many poly-Q containing proteins). Huntingtin fragments, recognized to form amyloid-like inclusions, have also been excluded from the analysis due to severe uncertainty in determining the length of the fragments [27], [28] The two databases, listing the names of the proteins, their sizes, their associated pathologies and references are reported in Tables S1 and S2, respectively (see supplementary information on-line).

**Figure 1:**
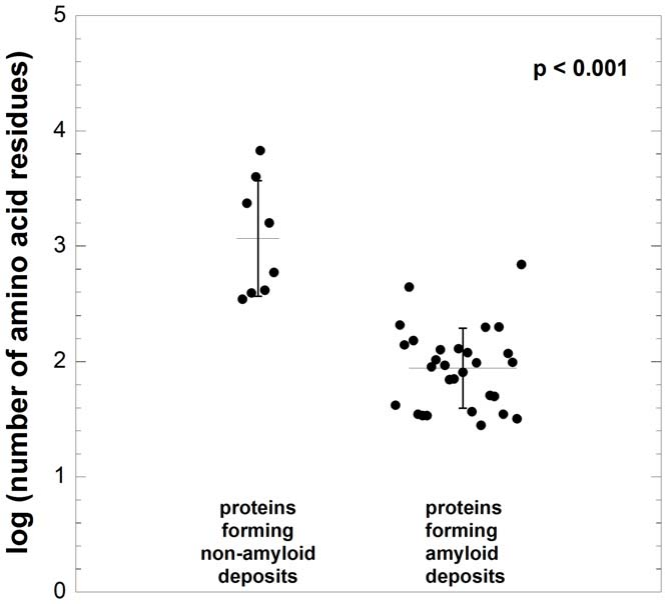
Size distributions of proteins reported to form non-amyloid deposits in human pathology (left) and of proteins described to form extracellular amyloid fibrils or intracellular inclusions with amyloid-like characteristics in human disease (right). Each data point represents one peptide or protein with its size described as a logarithm of the number of amino acid residues (*y* axis). A list of all the proteins reported in the graph and of their sizes is shown in Tables S1 and S2. The scatter of data points on the *x* axis has no meaning and it has been introduced to separate the data points in the graph. The horizontal and vertical lines indicate the mean values and the associated standard deviations for both populations. The size distributions are significantly different (p<0.001).

Fig. 1 shows the sizes, reported as the logarithm of the number of amino acid residues, of all the proteins belonging to the first and second group. The size distributions of the two groups of proteins appear to be well separated (Fig. 1). The proteins reported to form non-amyloid deposits are, on average, bigger in size, with lengths ranging from 345 to a few thousands of residues (Fig. 1, Table S2). By contrast, amyloid-forming proteins are typically smaller than 250 residues, with tau and lactoferrin representing the only exceptions in a group of 25 cases (Fig. 1, Table S1). The size distributions of the two populations of proteins are significantly different (p<0.001).

In our analysis the immunoglobulin light chain has been assigned to the group of amyloid-forming proteins because of the prevalence of light chain amyloidosis with respect to light chain deposition disease. Along the same lines, the immunoglobulin heavy chain has been assigned to the non-amyloid database, due to the prevalence of heavy chain deposition disease with respect to heavy chain amyloidosis. Serpins and hemoglobin, which are associated with serpinopathies and cell sickle anemia, respectively, have been excluded from the analysis because they both form fibrillar aggregates that are distinct from amyloid, yet highly structured. Remarkably, however, the addition of hemoglobin and serpin to either database, and the concomitant transfer of the immunoglobulin light chain and heavy chain from their currently assigned databases to the other, does not compromise the statistical significance of the difference observed for the two groups of proteins (p<0.01 in all cases). This highlights the robustness of the statistical analysis and indicates that the observed difference between the two groups of proteins does not depend on the assignment of these few “ambiguous” cases.

### YHKB converts into a partially unfolded, potentially amyloidogenic state at low Ph

We then investigated the aggregation process *in vitro* of the 486-residue YHKB. The size of this protein falls within the region of overlap of the size distributions of proteins forming amyloid and non-amyloid deposits in pathology [log(486) = 2.68]. YHKB was used as a sample protein. The study of this protein was not aimed at demonstrating that none of the large proteins forms amyloid-like fibrils, but at investigating the behavior of an intermediate size protein under conditions known to lead to amyloid fibril formation in vitro. We first determined whether this protein forms amyloid-like fibrils under conditions of low pH in the presence of salts, which are known to be effective in promoting amyloid formation not just from proteins normally associated with amyloid diseases [13]–[16], [18], but also from proteins with no link to disease [9], [11], [17], [20]–[22].

**Figure 2:**
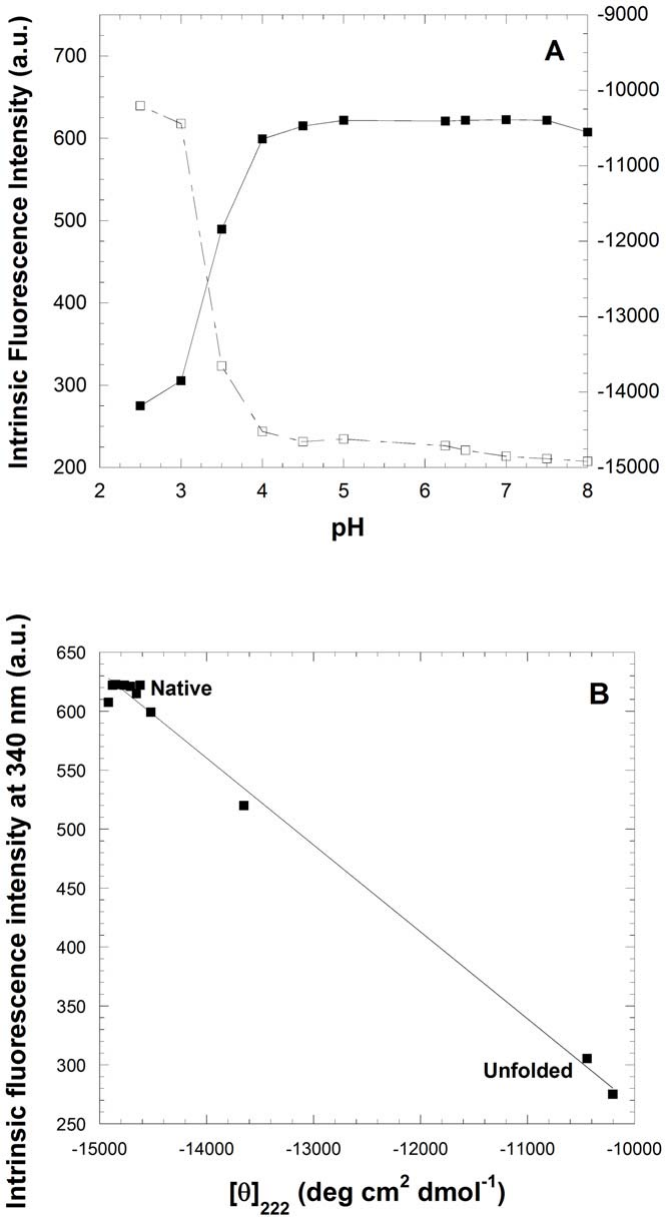
H-induced unfolding of YHKB. (A) pH-induced unfolding of YHKB monitored by the fluorescence emission intensity at the maximum wavelength (▪) and [θ]_222_ (□). (B) Spectral diagram of pH-induced equilibrium unfolding of YHKB, reporting on F_340_ versus [θ]_222_.

Amyloid fibril formation by globular proteins generally requires the presence of a partially unfolded state [8], [29]. Intrinsic fluorescence and far-UV CD spectroscopy were used to monitor possible conformational changes of the protein as the pH is decreased from neutral to acid values in the absence of salts. Both spectroscopic probes monitored substantial changes as the pH is reduced from 4 to 3, with a single sharp transition (Fig. 2A). The pH dependence of ANS fluorescence also showed a similar transition within the same pH range (data not shown). The spectral diagram reporting intrinsic fluorescence at 340 nm (F_340_) versus the mean residue ellipticity at 222 nm ([θ]_222_) possesses only one linear part (Fig. 2B). This indicates the existence of one transition in the pH titration of the enzyme and suggests that a two-state model is a good approximation to describe the pH unfolding of the enzyme under the investigated pH range.

**Figure 3:**
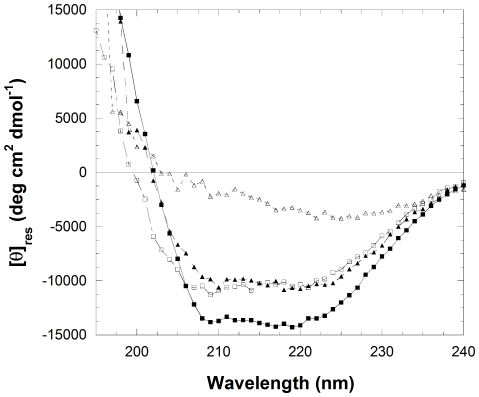
Far-UV CD spectra of native YHKB in 50 mM phosphate buffer, pH 7.0 (▪), in 20 mM TFA, pH 1.7 without salts (□), in 20 mM TFA, pH 1.7 with 360 mM NaCl after 5 min (▴) and under the same conditions after 120 min (Δ).

A comparison between the far-UV CD spectra acquired at pH 7.0 and 1.7 in the absence of salts indicated a significant conformational change following the pH decrease (Fig. 3). However, it is clear that the CD spectrum of the pH-denatured state is not typical of a fully unfolded state and rather indicates the persistence of significant secondary structure (Fig. 3). Overall, the pH-denatured state of YHKB is a partially unfolded state and thus a good candidate for amyloid fibril formation by this enzyme.

### Acid-denatured YHKB converts into aggregates with β-sheet structure, weak ThT- and CR-binding

Addition of salts to samples containing proteins denatured at low pH is known to accelerate aggregation dramatically [30], [31]. Hence, to assess whether the acid-denatured state of YHKB is amyloidogenic, YHKB was incubated in 20 mM TFA, pH 1.7, in the presence of various concentrations of NaCl ranging from 360 to 810 mM. The far-UV CD spectrum acquired after 5 minute incubation at pH 1.7 with 360 mM NaCl showed subtle, yet significant, changes with respect to that acquired before salt addition (Fig. 3). The change became more and more apparent with time and the spectrum acquired under the same conditions after 2 hours contained a low CD signal and a major negative peak in the 220–230 nm region (Fig. 3). Despite this peak being red-shifted with respect to the canonical β-sheet profile, such a feature is typical of large aggregates containing β-sheet structure [12], [32], [33]. The red-shift of the peak wavelength and the decrease of signal intensity arise from the *differential absorption flattening effect* arising from heterogeneous samples in which solid state material is suspended in solution [34]. Overall, the far-UV CD analysis indicated the presence of β-structured aggregates at early time points following incubation under the conditions employed here. Similar results were obtained for higher NaCl concentrations investigated, such as 540 and 810 mM, with the rate of formation of β-structured aggregates increasing with NaCl concentration (data not shown).

**Figure 4:**
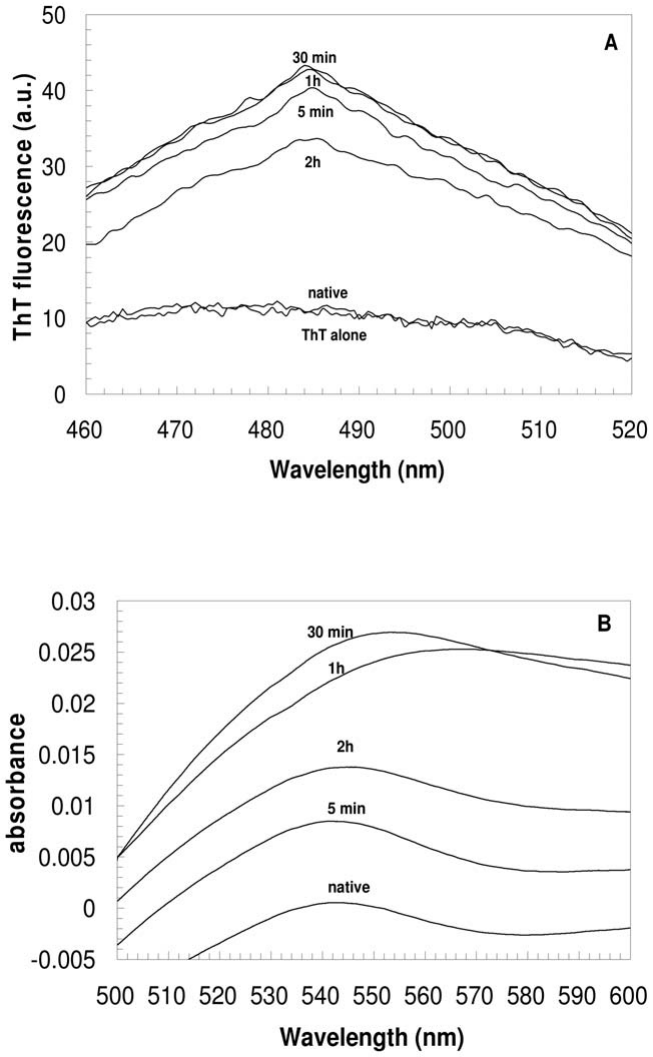
ThT and CR binding of YHKB aggregates at low pH. (A) ThT fluorescence emission spectra obtained after addition of YHKB samples pre-incubated at pH 1.7, 360 mM NaCl for 5, 30, 60 and 120 minutes. All spectra were obtained after subtraction of the corresponding spectra obtained with ThT in the absence of YHKB. The spectrum of ThT alone and that obtained after addition of the native protein are also shown for comparison. (B) *Difference* absorbance spectra of CR obtained with YHKB samples pre-incubated as described in panel (A). In each case, the difference spectrum was obtained by subtracting the spectra acquired for the protein alone and for CR alone from the spectrum recorded for the protein in the presence of CR.

Protein samples incubated for various lengths of time at pH 1.7 in the presence of 360 mM NaCl were also analyzed using the ThT fluorescence and the CR optical absorption assays. ThT is widely used as a marker of amyloid fibril formation *in vitro*, manifesting a strong increase in fluorescence intensity in the presence of amyloid fibrils [6]. The fluorescence emission spectra of ThT, obtained after the addition of YHKB pre-incubated for 5, 30, 60 and 120 minutes under these conditions, featured a weak, albeit significant, increase of fluorescence with respect to those obtained from either ThT alone or ThT in the presence of the native protein (Fig. 4A).

CR is also widely used to reveal the presence of amyloid fibrils. It binds to the fibrils with its absorption maximum shifting from 490 to 540 nm [6]. The *difference* spectra of CR obtained using the protein samples after 5, 30, 60 and 120 minutes of pre-incubation showed a maximum at ca. 540 nm (Fig. 4B). The height of the peak in the difference spectrum was significantly higher than that obtained using the native protein, indicating formation of aggregates (Fig. 4B). In both ThT and CR assays the maximum effect was obtained after 30–60 minutes.

### The aggregates formed by YHKB at low pH appear to be morphologically amorphous

**Figure 5:**
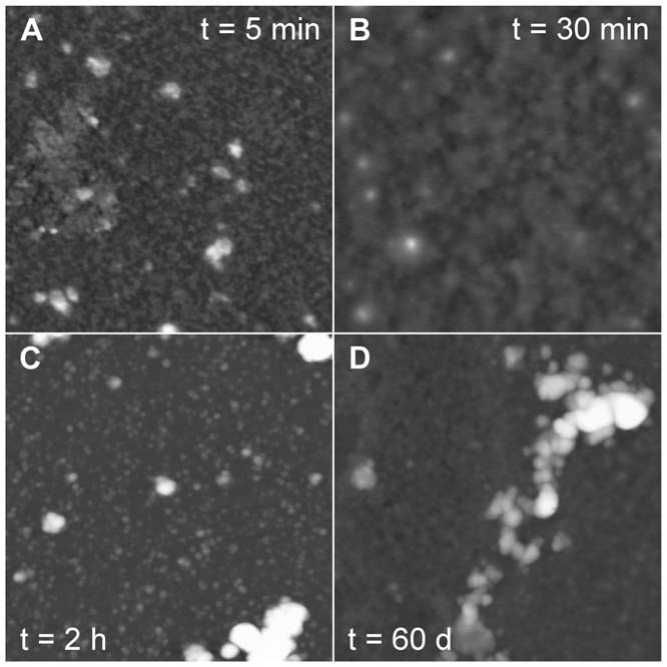
TM-AFM images (height data) of YHKB aggregates obtained in 20 mM TFA, 360 mM NaCl, pH 1.7. In each frame the corresponding aggregation time t is reported. Scan size 1 µm; Z range 20 nm (A–C) and 30 nm (D).

The YHKB samples aged at pH 1.7 in 360 mM NaCl were also analyzed using TM-AFM. The aggregates appeared to be amorphous after 5 and 30 minutes (Fig. 5A, B). After 2 h incubation, small structures with a height between 3 and 7 nm were found to coexist with assemblies of aggregates of variable size and height between 10 and 50 nm (Fig. 5C). It is well known that amyloid-like fibrils may form after prolonged incubation whereas oligomers with β-sheet structure and a weak ability to bind ThT and CR may appear much earlier [1]. AFM images were then acquired after 60 days incubation under the same conditions. However, fibrils were not yet apparent in the sample at this time: large clusters of aggregates of variable size were observed, along with smaller species with undefined morphology (Fig. 5D).

### YHKB converts into a partially unfolded, potentially amyloidogenic state in TFE

**Figure 6:**
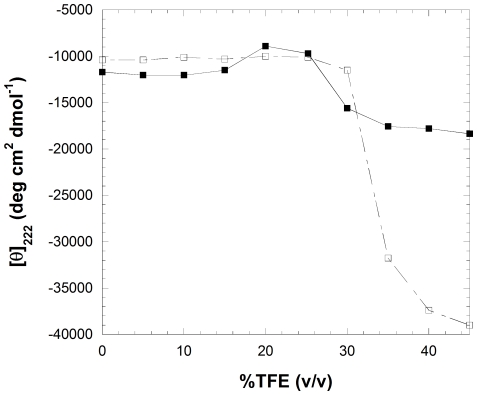
TFE-induced unfolding of YHKB monitored by [θ]_222_ in the absence (▪) and/or presence (□) of 1 mM α-cyclodextrin.

Solution conditions containing moderate concentrations of TFE have also been widely used to form amyloid-like fibrils by both disease-related and disease-unrelated proteins [10], [12], [19], [21]–[23]. Similarly to the study performed at low pH, we first acquired far-UV CD and fluorescence spectra in the presence of various concentrations of TFE, ranging from 0 to 45% (v/v) at pH 5.5. The intrinsic fluorescence of YHKB was found to decay rapidly with TFE concentration, due to the ability of TFE to quench dramatically tryptophan and tyrosine fluorescence. By contrast, the far-UV CD spectra were found to be similar from 0 to 15% (v/v) TFE, decrease in intensity at 20–26% (v/v) TFE and then increase dramatically to reach the appearance typical of an α-helical-enriched conformational state at TFE concentrations higher than 40%. The plot of [θ]_222_ versus TFE concentration allowed this behavior to be clearly observed (Fig. 6). The first transition causing a decrease of the CD signal at 20–26% (v/v) TFE is due to aggregation (see below), whereas the second transition is likely to arise from the conversion of the protein into a monomeric α-helical conformational state.

Cyclodextrins are potential protein folding aids [35]. Their effect on protein folding has been attributed to the ability of these cyclic compounds to suppress protein aggregation [36]. The TFE titration was therefore repeated in the presence of α-cyclodextrin (Fig. 6). The transition from the native to the aggregated state was abolished, whereas the second transition to a α-helical enriched state was found to occur at higher TFE concentrations (Fig. 6). This result indicates that the conversion of native YHKB into a TFE-induced partially unfolded state is, to a good approximation, a two-state process in the absence of aggregation.

### TFE-denatured YHKB converts into aggregates with β-sheet structure, weak ThT- and CR-binding

**Figure 7:**
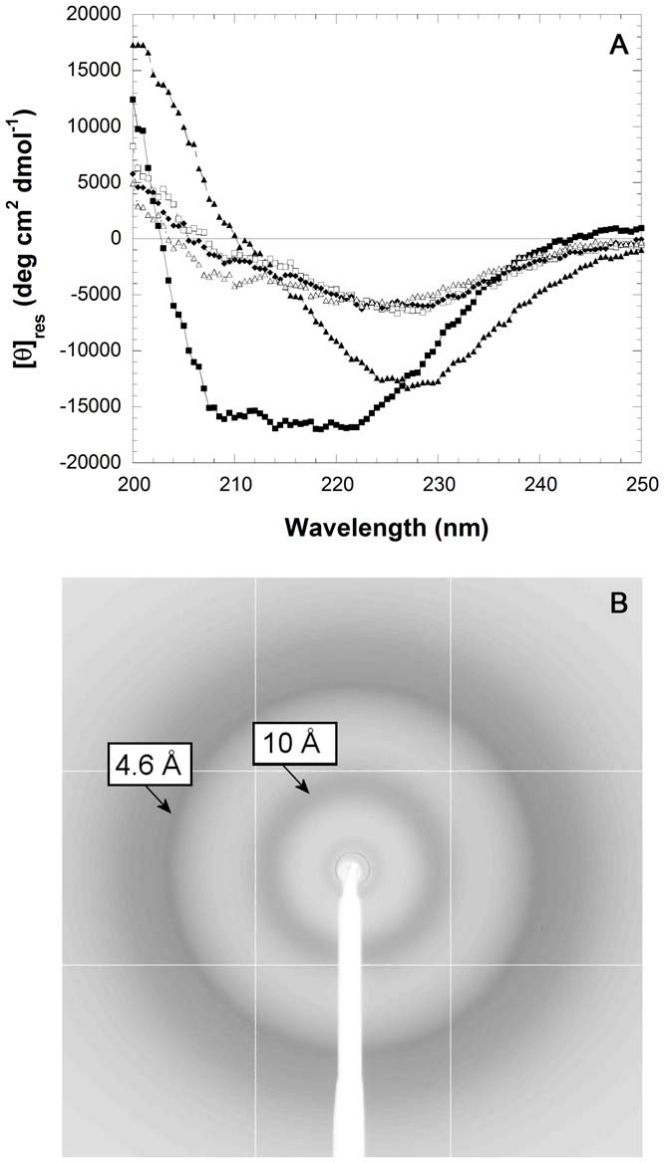
Far-UV CD and X-ray diffraction analysis of YHKB aggregates in TFE. (A) Far-UV CD spectra of native YHKB in 50 mM phosphate buffer, pH 7.0 (▪), in 50 mM acetate buffer, pH 5.5 with 30% (v/v) TFE after 5 minutes (▴), 30 minutes (□), 60 minutes (♦) and 120 minutes (Δ). (B) X-ray diffraction diagram of YHKB aggregates grown in 50 mM acetate buffer, pH 5.5 with 30% (v/v) TFE for 120 minutes. The diagram was collected on sedimented aggregates and shows the characteristic cross-β spacings at 4.6 and 10 Å.

To promote aggregation of YHKB, the protein was incubated at a concentration of 0.1 mg/ml in 50 mM acetate buffer, pH 5.5 and 25°C, in 30% (v/v) TFE. After 5 minutes the far-UV CD spectrum is typical of β-sheet structure, indicating the early accumulation of aggregates (Fig. 7A). Later, the far-UV CD spectrum decreased in intensity, suggesting the appearance of larger aggregates (Fig. 7A). The YHKB aggregates grown under the same conditions for 2 hours were also analysed by X-ray diffraction. The fibril X-ray diffraction pattern was consistent with the cross-β conformation (Fig. 7B). In particular, the diffraction pattern shows a strong reflection around 4.6 Å, corresponding to the hydrogen bonding distance between consecutive β-strands in β-sheets, and a strong reflection around 10 Å, corresponding to the face-to-face distance of the β-sheets in the aggregates (Fig. 7B).

**Figure 8:**
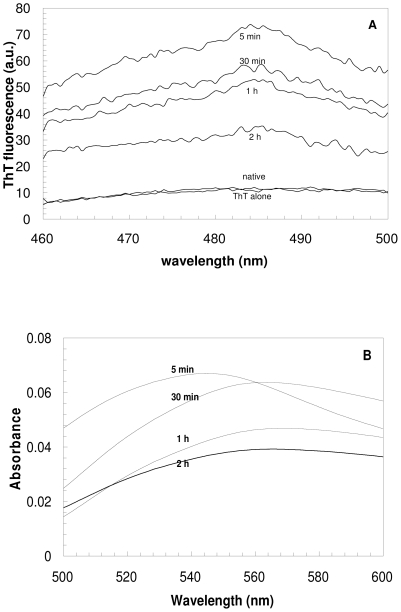
ThT and CR binding of YHKB aggregates in TFE. (A) ThT fluorescence emission spectra obtained after addition of YHKB samples pre-incubated at pH 5.5, 30% (v/v) TFE for 5, 30, 60 and 120 minutes. All spectra were obtained after subtraction of the corresponding spectra obtained with ThT in the absence of YHKB. The spectrum of ThT alone and the spectrum obtained after addition of the native protein are also shown for comparison. (B) *Difference* absorbance spectra of CR obtained with YHKB samples pre-incubated as described in panel (A). In each case, the difference spectrum was obtained by subtracting the spectra acquired for the protein alone and for CR alone from the spectrum recorded for the protein in the presence of CR.

The protein samples incubated under these conditions were also analyzed at various time intervals using the ThT and CR assays, similarly to the analysis performed at low pH. Significant increases in both the ThT fluorescence intensity and CR absorbance at 540 nm were observed after 5 minutes (Fig. 8). After 30 minutes both peaks decreased in intensity, with further decreases occurring after 60 and 120 minutes (Fig. 8).

### The aggregates formed by YHKB in the presence of TFE are not typical amyloid

**Figure 9:**
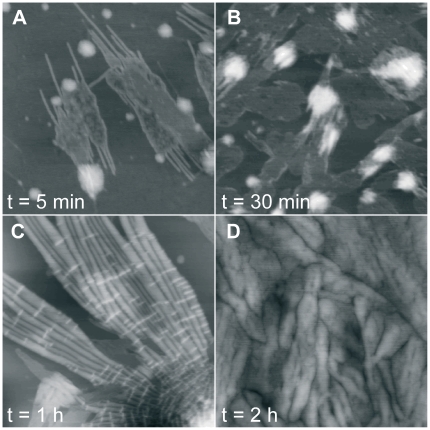
TM-AFM images (height data) of YHKB aggregates obtained in 30% (v/v) TFE, 50 mM acetate buffer, pH 5.5. In each frame the corresponding aggregation time t is reported. Scan size 1 µm; Z range 10 nm (A, B), 30 nm (C) and 20 nm (D).

The YHKB samples incubated at pH 5.5 in the presence of 30% (v/v) TFE were analyzed using TM-AFM. After 5 minutes the aggregates appeared to consist of thin fibrils with height between 2.2 and 2.7 nm, merging into large sheet-like structures (Fig. 9A). These species were found to coexist with globular structures with a height ranging from 5 to 10 nm (Fig. 9A). After 30 minutes large bundles with undefined morphology were present (Fig. 9B). After 1 hour incubation the aggregates appeared to consist of fibrils with a tubular morphology and a height of 7.0±0.2 nm (Fig. 9C). Nevertheless, these products had irregular width and appeared to branch into distinct tubular structures at many branching points. In addition, no regular twist was evident, as generally observed for amyloid fibrils. Another peculiar morphological feature was the presence of thinner fibril segments, 4.0±0.2 nm high, deposited on top of the tubular structures and running perpendicular to them (Fig. 9C). These structures were not found to be stable and reorganized further after 1 additional hour into a very tight network of irregular aggregates with variable width (Fig. 9D). Overall, similarly to the analysis performed at low pH, YHKB aggregation was very rapid in the presence of TFE, but resulted in structures that were unstable and morphologically different from regular amyloid-like fibrils. A difference between the two sets of conditions is that formation of aggregates with β-sheet structure and a significant, albeit weak, ability to bind ThT and CR was more rapid in the presence of TFE than that occurring at low pH.

### The aggregates formed by YHKB in the two sets of conditions cause cellular dysfunction

**Figure 10:**
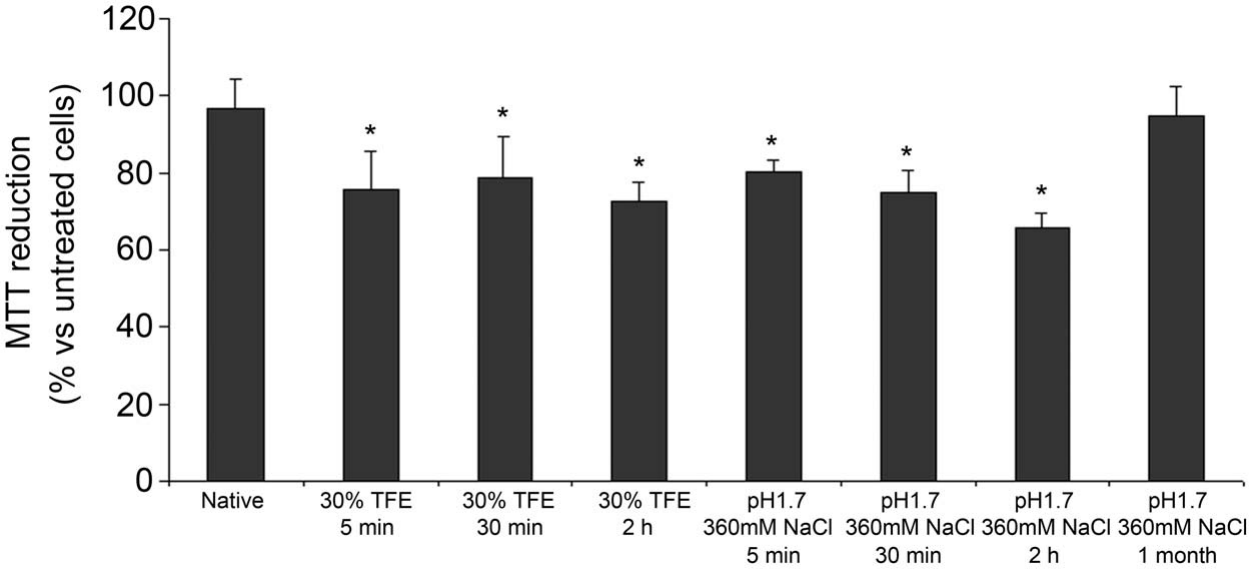
Cytotoxicity of YHKB aggregates. Cell viability was determined by the MTT reduction test in SH-SY5Y cells exposed to 2 µM native YHKB (bar 1), YHKB aggregates incubated for 5, 30 and 120 min in 30% (v/v) TFE at pH 5.5 (bars 2–4), YHKB aggregates incubated for 5 min, 30 min, 120 min and 1 month at pH 1.7, 360 mM NaCl (bars 5–8). The reported values are representative of two independent experiments, each carried out four times.

Both the aggregates formed at low pH without TFE and in the presence of 30% (v/v) TFE at pH 5.5 were transferred to a physiological medium and the resulting suspensions were added to the cell culture media of SH-SY5Y cells at a concentration of 2 µM (all concentrations refer to monomer concentration). The ability of the resulting aggregates to cause cellular dysfunction was assessed by performing the MTT reduction test [37]. Native YHKB did not cause any detectable decrease in the ability of cells to reduce MTT relative to untreated cells (Fig. 10). By contrast, the aggregates formed under both conditions studied here were found to cause a significant decrease in MTT reduction at all time points (Fig. 10). The toxicity of the aggregates pre-incubated at low pH was lost upon prolonged incubation under the same conditions (Fig. 10). We cannot assess whether the observed toxicity arises from the insoluble aggregates or soluble oligomers, either present in the sample or dissociating from the insoluble aggregates. This analysis would require further study which is beyond the scope of the present manuscript.

## Discussion

The analysis carried out here clearly indicates a remarkable difference between proteins forming amyloid deposits in human pathologies (or intracellular inclusions with related structural characteristics) and proteins forming deposits of a different nature in human diseases. Proteins forming non-amyloid deposits are bigger in size, suggesting a high tendency to aggregate coupled to a low propensity to form real amyloid fibrils for such proteins. To investigate the behavior of a sample protein with a size intermediate between the two populations, we have studied the *in vitro* aggregation process of YHKB, a fairly large protein having 486 residues and a molecular weight of 55 kDa. In order to force YHKB into amyloid-like aggregate formation, we have incubated this protein system in two distinct experimental conditions. These conditions have been previously found to be effective in converting globular proteins into amyloid-like fibrils, for both systems associated with amyloid-related pathologies [13]–[16], [18], [19] and model proteins with no link to protein deposition disease [9]–[12], [17], [20]–[23].

Neither condition was effective in converting YHKB into typical amyloid-like fibrils. The pH and TFE titrations indicated that YHKB unfolds into partially folded species under both conditions, indicating that the employed solution media were sufficient to promote partial unfolding of the protein, a circumstance that is ideal for amyloid fibril formation [29]. However, inspection with AFM of the protein samples aged in such media did not show a morphology typical of regular amyloid fibrils. At low pH in the presence of salts, the aggregates were either globular or amorphous. Even after prolonged incubation they appeared as large aggregates coexisting with small species with undefined morphology. By contrast, incubation of YHKB in the presence of TFE produced, after just five minutes, thin fibrils merging into wide sheets. After 1 hour, large tubular structures were present. These branched at many points, had an irregular width and no regular twist and were apparently surmounted by thinner fibrils running perpendicular to them. These structures were not stable and converted into a tight network of large structures of variable width after 1 additional hour. All these traits are not reminiscent of regular amyloid fibrils.

Despite the fact that the aggregates of YHKB did not have a morphology and stability typical of amyloid fibrils, YHKB aggregation appeared to be extremely rapid under both conditions. Species with β-sheet structure and a weak, yet significant, ability to bind ThT and CR were apparent after just 5 minutes incubation, indicating a significant tendency to form β-pleated aggregates in both media. Analysis carried out in TFE with X-ray diffraction confirmed the presence of cross-β structure. Such β-structured aggregates were also biologically harmful as their addition to the extracellular media of cultured human SH-SY5Y cells impaired cell viability to a significant extent.

Hence, YHKB can still aggregate under the conditions investigated here, as the protein is partially unfolded and the experimental media do not prevent formation of non-covalent bonds, which is an essential pre-requisite for protein aggregation to occur. Aggregation resulted in the formation of β-sheet structure, but not into well defined amyloid fibrils in this case, as it was found for many relatively small proteins under identical conditions [9]–[23]. It thus appears that the small propensity found for large proteins to form amyloid deposits *in vivo* under pathological conditions can be reproduced in the test tube, suggesting that such a behavior is an intrinsic characteristic of the big size of such proteins.

Can an explanation for such an inverse correlation between protein size and amyloid propensity be provided? The presence of a partially unfolded, multidomain protein does not allow the protein to form ordered amyloid fibrils as each single protein molecule can form a variety of contacts with other similarly unfolded molecules. This inherent self-assembly capacity may inhibit formation of regular, ordered and long structures and result rather into either amorphous structures, large and undefined sheets or branching tubular structures. The AFM images obtained in the presence of TFE after 1 hour are particular suggestive in this regard (Fig. 9C). The apparent tubular fibrils appear large in width, indicating that an extended portion of each protein molecule may form a variety of intermolecular contacts expanding the section area of the apparent fibril. Moreover, the tubular structures are branched at many points, raising the possibility that individual YHKB molecules can propagate more than one fibril type by multiple interactions with distinct YHKB molecules, each promoting formation of a new tubular structure. In addition, in the AFM images the tubular species seem to give rise to other fibrils, budding from them and appearing to be deposited on top of the former, possibly a manifestation of the three-dimensionality of fibril branching and propagation. However, despite such a high degree of polymorphism, the ability of forming aggregates with a cross-β structured core is still retained.

Overall, the ability to bind ThT and CR, albeit weak, the presence of a significant amount of β-sheet structure, as detected with far-UV CD and X-ray fiber diffraction, and the tendency to give rise to structures vaguely similar to fibrils suggest that the YHKB aggregates explored here have the germ of amyloid structure, but the large size of the protein prevents them from acquiring the order and regular structure typical of real amyloid fibrils.

## Supporting Information

Table S1 **Database reporting a list of peptides or proteins forming extracellular amyloid deposits or intracelullar inclusions with amyloid-like characteristics in human diseases.** The names of the proteins, their sizes, their associated pathologies and references are reported.
(DOC)

Table S2 **Database of peptides or proteins forming intracellular or extracellular non-amyloid deposits in human diseases.** The names of the proteins, their sizes, their associated pathologies and references are reported.
(DOC)
