# Supplementary material for: Large Proteins Have a Great Tendency to Aggregate but a Low Propensity to Form Amyloid Fibrils
Source: PLoS One. 2011 Jan 13;6(1):e16075. doi: 10.1371/journal.pone.0016075 (PMC3020945; doi:10.1371/journal.pone.0016075)
Supplement: Table S1 — Database reporting a list of peptides or proteins forming extracellular amyloid deposits or intracelullar inclusions with amyloid-like characteristics in human diseases. The names of the proteins, their sizes, their associated pathologies and references are reported. (DOC) [file pone.0016075.s001.doc]

| Table S1 – Peptides or proteins forming extracellular amyloid deposits or intracelullar inclusions with amyloid-like characteristics in human diseases ^a^ | | | | |
| --- | --- | --- | --- | --- |
| Full name of  peptide or protein | Short name of peptide or protein | Size  (number of residues) ^b^ | Diseases associated with the deposits  of the peptide or protein | Reference |
| amyloid β peptide | Aβ | 40 or 42 ^c^ | - Alzheimer’s disease  - Hereditary cerebral haemorrhage with amyloidosis | 1, 2 |
| prion protein | PrP | 208 | - Creutzfeldt-Jacob disease  - Gerstmann-Sträussler-Scheinker disease  - Fatal familial insomnia | 3, 4 |
| tau | τ | 352-441 ^c^ | - Pick’s disease  - Progressive supranuclear palsy  - Corticobasal degeneration  - Frontotemporal dementia with parkinsonism linked to chromos. 17  - Argyrophilic grain disease  - Tangle predominant dementia  - Guam Parkinson dementia complex | 5, 6 |
| ABri peptide | ABri | 34 | - Familial British dementia | 7, 8 |
| ADan peptide | ADan | 34 | - Familial Danish dementia | 9, 10, 11 |
| Immunoglobulin light chain variable domain ^e^ | - | ~ 90 | - AL amyloidosis | 12, 13 |
| Fragments of serum amyloid A protein | SAA | 76-104 ^c^ | - AA amyloidosis  - Familial Mediterranean fever | 14, 15 |
| transthyretin | TTR | 127 | - Senile systemic amyloidosis  - Familial amyloidotic polyneuropathy  - Familial amyloid cardiomyopathy  - Leptomeningeal amyloidosis  - Carpal tunnel syndrome  - Vitreous opacity | 16, 17 |
| β2-microglobulin | β2-m | 99 | - Hemodialysis-related amyloidosis | 18, 19 |
| N-terminal fragments of apolipoprotein AI | apoAI | 73-94 ^c^ | - ApoAI amyloidosis | 20, 21, 22, 23, 24 |
| C-terminally extended apolipoprotein AII | apoAII | 98 | - ApoAII amyloidosis | 25 |
| N-terminal fragment of apolipoprotein AIV | apoAIV | ~ 70 | - ApoAIV amyloidosis | 26, 27 |
| Fragments of gelsolin | - | 53 or 71 ^c^ | - Finnish hereditary amyloidosis | 28, 29 |
| lysozyme | - | 130 | - Lysozyme amyloidosis | 30 |
| fragments of fibrinogen α-chain | - | 45-81 ^c^ | - Fibrinogen amyloidosis | 31, 32, 33, 34, 35, 36 |
| cystatin C | - | 120 | - Hereditary cerebral hemorrhage with amyloidosis, Icelandic type | 37, 38 |
| islet amyloid polypeptide | IAPP | 37 | - Type II diabetes | 39, 40 |
| Calcitonin | - | 32 | - Medullary carcinoma of the thyroid | 41, 42 |
| Atrial natriuretic factor | ANF | 28 | - Atrial amyloidosis | 43, 44 |
| Prolactin | PRL | 199 | - Pituitary prolactinoma | 45 |
| Insulin | - | 51 ^f^ | - Injection-localized amyloidosis | 46 |
| Medin | - | 50 | - Aortic medial amyloidosis | 47, 48 |
| Lactotransferrin | lactoferrin | 692 | - Localised amyloidosis of the seminal vescicle  - Familial subepithelial corneal amyloidosis  - Corneal amylodosis associated with trichiasis | 49, 50, 51 |
| Odontogenic amenoblast-associated protein | ODAM | 110-118 ^c^ | - Calcifying epithelial odontogenic tumors | 52, 53 |
| **Pulmonary surfactant-associated protein C** | SP-C | 35 | - Pulmonary alveolar proteinosis | 54 |
| **^a^ The peptides and proteins listed in the table include cases reported to form either extracellular amyloid deposits or intracellular inclusions with characteristics reminiscent of amyloid structures.**  **^b^ Data refer to the processed polypeptide chains that deposit into aggregates, not to the precursor proteins.**  **^c^ Only the longest isoform or fragment has been selected for the analysis reported in the main text (Fig. 1).**  **^d^ minimal length of the poly-Q stretch found to promote aggregation of the poly-Q-bearing protein.**  **^e^ These proteins have also been reported to form non-amyloid deposits in some specific diseases, but have been assigned to the group of amyloid-forming proteins due to the prevalence of amyloid-related diseases.**  **^f^ Calculated by considering both the A and B chains, which are linked by a disulphide bridge** | | | | |

**References**

1. Divry, P. (1934). *J. Belge. Neurol. Psychiatry* **34**, 197-201.
2. [Masters, C. L](http://www.ncbi.nlm.nih.gov/pubmed?term="Masters CL"%5BAuthor%5D)., [Simms, G](http://www.ncbi.nlm.nih.gov/pubmed?term="Simms G"%5BAuthor%5D)., [Weinman, N. A](http://www.ncbi.nlm.nih.gov/pubmed?term="Weinman NA"%5BAuthor%5D)., [Multhaup, G](http://www.ncbi.nlm.nih.gov/pubmed?term="Multhaup G"%5BAuthor%5D)., [McDonald, B. L](http://www.ncbi.nlm.nih.gov/pubmed?term="McDonald BL"%5BAuthor%5D). and [Beyreuther, K](http://www.ncbi.nlm.nih.gov/pubmed?term="Beyreuther K"%5BAuthor%5D). (1985) [*Proc. Natl. Acad. Sci. U S*](javascript:AL_get(this, 'jour', 'Proc Natl %0D%0AAcad Sci U S A.');) *A* **82**, 4245-4249.
3. [Chou, S. M](http://www.ncbi.nlm.nih.gov/pubmed?term="Chou SM"%5BAuthor%5D). and [Martin, J. D](http://www.ncbi.nlm.nih.gov/pubmed?term="Martin JD"%5BAuthor%5D). (1971) [*Acta Neuropathol.*](javascript:AL_get(this, 'jour', 'Acta Neuropathol.');) **17**, 150-155.
4. Kitamoto, T., Tateishi, J., Tashima, I., Takeshita, I., Barry, R. A., DeArmond, S. J. and Prusiner, S. B. (1986) *Ann. Neurol.* **20**, 204-208.
5. Berriman, J., Serpell, L. C., Oberg, K. A., Fink, A. L., Goedert, M. and Crowther, R. A. [(2003)](http://www.ncbi.nlm.nih.gov/pubmed/12853572) *Proc. Natl. Acad. Sci. U S A* **100**, 9034-9038.
6. Dickson, D. W. (2009) *Int. J. Clin. Exp. Pathol.* **3**, 1-23.
7. Worster-Drought, C., Greenfield, J. G. and McMenemey W.H. (1940) Brain **63**, 237-254.
8. Vidal, R., Frangione, B., Rostagno, A., Mead, S., Revesz, T., Plant, G. and Ghiso, J. (1999) *Nature* **399**, 776-781.
9. Strömgrem, E., Dalby, A., Dalby, M. and Ranheim, B. (1970) Acta Neurol. Scand. **46**, 97–98.
10. Strömgrem, E. (1981) *Handbook of Clinical Neurology*, Vinken, P. J. and Bruyn, G. W. Eds, Elsevier, Amsterdam.
11. [Vidal, R](http://www.ncbi.nlm.nih.gov/pubmed?term="Vidal R"%5BAuthor%5D)., [Revesz, T](http://www.ncbi.nlm.nih.gov/pubmed?term="Revesz T"%5BAuthor%5D)., [Rostagno, A](http://www.ncbi.nlm.nih.gov/pubmed?term="Rostagno A"%5BAuthor%5D)., [Kim, E](http://www.ncbi.nlm.nih.gov/pubmed?term="Kim E"%5BAuthor%5D)., [Holton, J. L](http://www.ncbi.nlm.nih.gov/pubmed?term="Holton JL"%5BAuthor%5D)., [Bek, T](http://www.ncbi.nlm.nih.gov/pubmed?term="Bek T"%5BAuthor%5D)., [Bojsen-Møller, M](http://www.ncbi.nlm.nih.gov/pubmed?term="Bojsen-Møller M"%5BAuthor%5D)., [Braendgaard, H](http://www.ncbi.nlm.nih.gov/pubmed?term="Braendgaard H"%5BAuthor%5D)., [Plant, G](http://www.ncbi.nlm.nih.gov/pubmed?term="Plant G"%5BAuthor%5D)., [Ghiso, J](http://www.ncbi.nlm.nih.gov/pubmed?term="Ghiso J"%5BAuthor%5D). and [Frangione, B](http://www.ncbi.nlm.nih.gov/pubmed?term="Frangione B"%5BAuthor%5D). (2000) [*Proc. Natl. Acad. Sci. U S A*](javascript:AL_get(this, 'jour', 'Proc Natl %0D%0AAcad Sci U S A.');) **97**, 4920-4925.
12. Glenner, G. G., Harbaugh, J., Ohma, J. I., Harada, M. and Cuatrecasas, P. (1970) *Biochem. Biophys. Re.s Commun.* **41**, 1287-1289.
13. Glenner, G. G., Terry, W., Harada, M., Isersky, C. and Page, D. (1971) *Science* **172**, 1150-1151.
14. Johnston, R., (1947) *Jefferson-Hillman Hosp. Bull.* **1**, 110-114.
15. [Benditt, E. P](http://www.ncbi.nlm.nih.gov/pubmed?term="Benditt EP"%5BAuthor%5D). and [Eriksen, N](http://www.ncbi.nlm.nih.gov/pubmed?term="Eriksen N"%5BAuthor%5D). (1971) [*Am. J. Pathol.*](javascript:AL_get(this, 'jour', 'Am J Pathol.');) **65**, 231-252.
16. Andrade, C. (1952) *Brain* **75**, 408-427.
17. [Rapezzi, C](http://www.ncbi.nlm.nih.gov/pubmed?term="Rapezzi C"%5BAuthor%5D)., [Quarta, C. C](http://www.ncbi.nlm.nih.gov/pubmed?term="Quarta CC"%5BAuthor%5D)., [Riva, L](http://www.ncbi.nlm.nih.gov/pubmed?term="Riva L"%5BAuthor%5D)., [Longhi, S](http://www.ncbi.nlm.nih.gov/pubmed?term="Longhi S"%5BAuthor%5D)., [Gallelli, I](http://www.ncbi.nlm.nih.gov/pubmed?term="Gallelli I"%5BAuthor%5D)., [Lorenzini, M](http://www.ncbi.nlm.nih.gov/pubmed?term="Lorenzini M"%5BAuthor%5D)., [Ciliberti, P](http://www.ncbi.nlm.nih.gov/pubmed?term="Ciliberti P"%5BAuthor%5D)., [Biagini, E](http://www.ncbi.nlm.nih.gov/pubmed?term="Biagini E"%5BAuthor%5D)., [Salvi, F](http://www.ncbi.nlm.nih.gov/pubmed?term="Salvi F"%5BAuthor%5D). and Branzi, A. (2010) [*Nat. Rev. Cardiol.*](javascript:AL_get(this, 'jour', 'Nat Rev %0D%0ACardiol.');) (published online)
18. [Charra, B](http://www.ncbi.nlm.nih.gov/pubmed?term="Charra B"%5BAuthor%5D)., [Calemard, E](http://www.ncbi.nlm.nih.gov/pubmed?term="Calemard E"%5BAuthor%5D)., Uzan, M., [Terrat, J. C](http://www.ncbi.nlm.nih.gov/pubmed?term="Terrat JC"%5BAuthor%5D)., [Vanel, T](http://www.ncbi.nlm.nih.gov/pubmed?term="Vanel T"%5BAuthor%5D). and [Laurent, G](http://www.ncbi.nlm.nih.gov/pubmed?term="Laurent G"%5BAuthor%5D). (1985) [*Proc. Eur. Dial. Transplant Assoc. Eur. Ren. Assoc.*](javascript:AL_get(this, 'jour', 'Proc Eur %0D%0ADial Transplant Assoc Eur Ren Assoc.');) **21**, 291-295.
19. [Gorevic, P. D](http://www.ncbi.nlm.nih.gov/pubmed?term="Gorevic PD"%5BAuthor%5D)., [Munoz, P. C](http://www.ncbi.nlm.nih.gov/pubmed?term="Munoz PC"%5BAuthor%5D)., [Casey, T. T](http://www.ncbi.nlm.nih.gov/pubmed?term="Casey TT"%5BAuthor%5D)., DiRaimondo, C. R., [Stone, W. J](http://www.ncbi.nlm.nih.gov/pubmed?term="Stone WJ"%5BAuthor%5D)., [Prelli, F. C](http://www.ncbi.nlm.nih.gov/pubmed?term="Prelli FC"%5BAuthor%5D)., [Rodrigues, M. M](http://www.ncbi.nlm.nih.gov/pubmed?term="Rodrigues MM"%5BAuthor%5D)., [Poulik, M. D](http://www.ncbi.nlm.nih.gov/pubmed?term="Poulik MD"%5BAuthor%5D). and [Frangione, B](http://www.ncbi.nlm.nih.gov/pubmed?term="Frangione B"%5BAuthor%5D). (1986) [*Proc. Natl. Acad. Sci. U S A*](javascript:AL_get(this, 'jour', 'Proc Natl %0D%0AAcad Sci U S A.');) **83**, 7908-7912.
20. [Van Allen, M. W](http://www.ncbi.nlm.nih.gov/pubmed?term="Van Allen MW"%5BAuthor%5D)., [Frohlich, J. A](http://www.ncbi.nlm.nih.gov/pubmed?term="Frohlich JA"%5BAuthor%5D). and [Davis, J. R](http://www.ncbi.nlm.nih.gov/pubmed?term="Davis JR"%5BAuthor%5D). (1969) *Neurology* **19**, 10-25.
21. [Nichols, W. C](http://www.ncbi.nlm.nih.gov/pubmed?term="Nichols WC"%5BAuthor%5D)., [Dwulet, F. E](http://www.ncbi.nlm.nih.gov/pubmed?term="Dwulet FE"%5BAuthor%5D)., [Liepnieks, J](http://www.ncbi.nlm.nih.gov/pubmed?term="Liepnieks J"%5BAuthor%5D)., Benson, M. D. (1988) [*Biochem. Biophys. Res. Commun.*](javascript:AL_get(this, 'jour', 'Biochem %0D%0ABiophys Res Commun.');) **156**, 762-768.
22. [Soutar, A. K](http://www.ncbi.nlm.nih.gov/pubmed?term="Soutar AK"%5BAuthor%5D)., [Hawkins, P. N](http://www.ncbi.nlm.nih.gov/pubmed?term="Hawkins PN"%5BAuthor%5D)., [Vigushin, D. M](http://www.ncbi.nlm.nih.gov/pubmed?term="Vigushin DM"%5BAuthor%5D)., [Tennent, G. A](http://www.ncbi.nlm.nih.gov/pubmed?term="Tennent GA"%5BAuthor%5D)., [Booth, S. E](http://www.ncbi.nlm.nih.gov/pubmed?term="Booth SE"%5BAuthor%5D)., [Hutton, T](http://www.ncbi.nlm.nih.gov/pubmed?term="Hutton T"%5BAuthor%5D)., [Nguyen, O](http://www.ncbi.nlm.nih.gov/pubmed?term="Nguyen O"%5BAuthor%5D)., [Totty, N. F](http://www.ncbi.nlm.nih.gov/pubmed?term="Totty NF"%5BAuthor%5D)., [Feest, T. G](http://www.ncbi.nlm.nih.gov/pubmed?term="Feest TG"%5BAuthor%5D)., [Hsuan, J. J](http://www.ncbi.nlm.nih.gov/pubmed?term="Hsuan JJ"%5BAuthor%5D)., and Pepys, M. B. (1992) *Proc. Natl. Acad. Sci. USA* **89**, 7389-7393.
23. [Booth, D. R](http://www.ncbi.nlm.nih.gov/pubmed?term="Booth DR"%5BAuthor%5D)., [Tan, S. Y](http://www.ncbi.nlm.nih.gov/pubmed?term="Tan SY"%5BAuthor%5D)., [Booth, S. E](http://www.ncbi.nlm.nih.gov/pubmed?term="Booth SE"%5BAuthor%5D)., [Hsuan, J. J](http://www.ncbi.nlm.nih.gov/pubmed?term="Hsuan JJ"%5BAuthor%5D)., [Totty, N. F](http://www.ncbi.nlm.nih.gov/pubmed?term="Totty NF"%5BAuthor%5D)., [Nguyen, O](http://www.ncbi.nlm.nih.gov/pubmed?term="Nguyen O"%5BAuthor%5D)., [Hutton, T](http://www.ncbi.nlm.nih.gov/pubmed?term="Hutton T"%5BAuthor%5D)., [Vigushin, D. M](http://www.ncbi.nlm.nih.gov/pubmed?term="Vigushin DM"%5BAuthor%5D)., [Tennent, G. A](http://www.ncbi.nlm.nih.gov/pubmed?term="Tennent GA"%5BAuthor%5D)., [Hutchinson, W. L](http://www.ncbi.nlm.nih.gov/pubmed?term="Hutchinson WL"%5BAuthor%5D)., Thomson, N., Soutar, A. K., Hawkins, P.N. and Pepys M.B. (1995) *QJM* **88**, 695-702.
24. [Booth, D. R](http://www.ncbi.nlm.nih.gov/pubmed?term="Booth DR"%5BAuthor%5D)., [Tan, S. Y](http://www.ncbi.nlm.nih.gov/pubmed?term="Tan SY"%5BAuthor%5D)., [Booth, S. E](http://www.ncbi.nlm.nih.gov/pubmed?term="Booth SE"%5BAuthor%5D)., [Tennent, G. A](http://www.ncbi.nlm.nih.gov/pubmed?term="Tennent GA"%5BAuthor%5D)., [Hutchinson, W. L](http://www.ncbi.nlm.nih.gov/pubmed?term="Hutchinson WL"%5BAuthor%5D)., [Hsuan, J. J](http://www.ncbi.nlm.nih.gov/pubmed?term="Hsuan JJ"%5BAuthor%5D)., [Totty, N. F](http://www.ncbi.nlm.nih.gov/pubmed?term="Totty NF"%5BAuthor%5D)., [Truong, O](http://www.ncbi.nlm.nih.gov/pubmed?term="Truong O"%5BAuthor%5D)., [Soutar, A. K](http://www.ncbi.nlm.nih.gov/pubmed?term="Soutar AK"%5BAuthor%5D)., [Hawkins, P. N](http://www.ncbi.nlm.nih.gov/pubmed?term="Hawkins PN"%5BAuthor%5D)., [Bruguera, M](http://www.ncbi.nlm.nih.gov/pubmed?term="Bruguera M"%5BAuthor%5D)., [Caballería, J](http://www.ncbi.nlm.nih.gov/pubmed?term="Caballería J"%5BAuthor%5D)., [Solé, M](http://www.ncbi.nlm.nih.gov/pubmed?term="Solé M"%5BAuthor%5D)., [Campistol, J.M](http://www.ncbi.nlm.nih.gov/pubmed?term="Campistol JM"%5BAuthor%5D). and [Pepys, M. B](http://www.ncbi.nlm.nih.gov/pubmed?term="Pepys MB"%5BAuthor%5D). (1996) [*J. Clin. Invest.*](javascript:AL_get(this, 'jour', 'J Clin %0D%0AInvest.');) **97**, 2714-2721.
25. [Benson, M. D](http://www.ncbi.nlm.nih.gov/pubmed?term="Benson MD"%5BAuthor%5D)., [Liepnieks, J. J](http://www.ncbi.nlm.nih.gov/pubmed?term="Liepnieks JJ"%5BAuthor%5D)., [Yazaki, M](http://www.ncbi.nlm.nih.gov/pubmed?term="Yazaki M"%5BAuthor%5D)., [Yamashita, T](http://www.ncbi.nlm.nih.gov/pubmed?term="Yamashita T"%5BAuthor%5D)., [Hamidi Asl, K](http://www.ncbi.nlm.nih.gov/pubmed?term="Hamidi Asl K"%5BAuthor%5D)., [Guenther, B](http://www.ncbi.nlm.nih.gov/pubmed?term="Guenther B"%5BAuthor%5D). and [Kluve-Beckerman, B](http://www.ncbi.nlm.nih.gov/pubmed?term="Kluve-Beckerman B"%5BAuthor%5D). (2001) [*Genomics*](http://www.sciencedirect.com/science/journal/08887543) **72**, 272-277.


    References and further reading may be available for this article. To view references and further reading you must [purchase](http://www.sciencedirect.com/science?_ob=ArticleURL&_udi=B6WG1-458NN02-5K&_user=10&_coverDate=03%2F15%2F2001&_rdoc=1&_fmt=full&_orig=search&_cdi=6809&_sort=d&_docanchor=&view=c&_searchStrId=1345064478&_rerunOrigin=google&_acct=C000050221&_version=1&_urlVersion=0&_userid=10&md5=108396025c991272e4e0d0832309de28) this article.
26. Bergström, J., Murphy, C., Eulitz, M., Weiss, D. T., Westermark, G. T., Solomon, A. and Westermark, P. (2001) *Biochem. Biophys. Res. Commun.* **285**, 903-908.
27. Bergström, J., Murphy, C. L., Weiss, D. T., Solomon, A., Sletten, K., Hellman, U. and Westermark, P. (2004) *Lab. Invest.* **84**, 981-988.
28. [Meretoja, J](http://www.ncbi.nlm.nih.gov/pubmed?term="Meretoja J"%5BAuthor%5D). (1969) [*Ann. Clin. Res.*](javascript:AL_get(this, 'jour', 'Ann Clin %0D%0ARes.');) **1**, 314-324.
29. Maury, C. P. (1991) *J. Clin. Invest.* **87**, 1195-1199.
30. [Pepys, M. B](http://www.ncbi.nlm.nih.gov/pubmed?term="Pepys MB"%5BAuthor%5D)., [Hawkins, P. N](http://www.ncbi.nlm.nih.gov/pubmed?term="Hawkins PN"%5BAuthor%5D)., [Booth, D. R](http://www.ncbi.nlm.nih.gov/pubmed?term="Booth DR"%5BAuthor%5D)., [Vigushin, D. M](http://www.ncbi.nlm.nih.gov/pubmed?term="Vigushin DM"%5BAuthor%5D)., [Tennent, G. A](http://www.ncbi.nlm.nih.gov/pubmed?term="Tennent GA"%5BAuthor%5D)., [Soutar, A. K](http://www.ncbi.nlm.nih.gov/pubmed?term="Soutar AK"%5BAuthor%5D)., [Totty, N](http://www.ncbi.nlm.nih.gov/pubmed?term="Totty N"%5BAuthor%5D)., [Nguyen, O](http://www.ncbi.nlm.nih.gov/pubmed?term="Nguyen O"%5BAuthor%5D)., [Blake, C. C](http://www.ncbi.nlm.nih.gov/pubmed?term="Blake CC"%5BAuthor%5D)., [Terry, C. J](http://www.ncbi.nlm.nih.gov/pubmed?term="Terry CJ"%5BAuthor%5D)., Feest, T. G., Zalin, A. M. and Hsuan, J. J. (1993) *Nature* **362**, 553-557.
31. Uemichi, T., Liepnieks, J. J. and Benson, M. D. (1994) *J. Clin. Invest.* **93**, 731-736.
32. Hamidi Asl, L., Liepnieks, J. J., Uemichi, T., Rebibou, J. M., Justrabo, E., Droz, D., Mousson, C., Chalopin, J. M., Benson, M. D., Delpech, M. and Grateau, G. (1997) *Blood* **90**, 4799-4805.
33. Uemichi, T., Liepnieks, J. J., Yamada, T., Gertz, M. A., Bang, N. and Benson, M. D. (1996) *Blood* **87**, 4197-4203.
34. [Kang, H. G](http://www.ncbi.nlm.nih.gov/pubmed?term="Kang HG"%5BAuthor%5D)., [Bybee, A](http://www.ncbi.nlm.nih.gov/pubmed?term="Bybee A"%5BAuthor%5D)., [Ha, I. S](http://www.ncbi.nlm.nih.gov/pubmed?term="Ha IS"%5BAuthor%5D)., [Park, M. S](http://www.ncbi.nlm.nih.gov/pubmed?term="Park MS"%5BAuthor%5D)., [Gilbertson, J. A](http://www.ncbi.nlm.nih.gov/pubmed?term="Gilbertson JA"%5BAuthor%5D)., [Cheong, H. I](http://www.ncbi.nlm.nih.gov/pubmed?term="Cheong HI"%5BAuthor%5D)., [Choi, Y](http://www.ncbi.nlm.nih.gov/pubmed?term="Choi Y"%5BAuthor%5D). and [Hawkins, P. N](http://www.ncbi.nlm.nih.gov/pubmed?term="Hawkins PN"%5BAuthor%5D). (2005) [*Kidney Int.*](javascript:AL_get(this, 'jour', 'Kidney %0D%0AInt.');) **68**, 1994-1998.
35. Benson, M. D. (2005) *Amyloid* **12**, 75-87.
36. [Benson, M. D](http://www.ncbi.nlm.nih.gov/pubmed?term="Benson MD"%5BAuthor%5D)., [Liepnieks, J](http://www.ncbi.nlm.nih.gov/pubmed?term="Liepnieks J"%5BAuthor%5D)., [Uemichi, T](http://www.ncbi.nlm.nih.gov/pubmed?term="Uemichi T"%5BAuthor%5D)., [Wheeler, G](http://www.ncbi.nlm.nih.gov/pubmed?term="Wheeler G"%5BAuthor%5D). and [Correa, R](http://www.ncbi.nlm.nih.gov/pubmed?term="Correa R"%5BAuthor%5D). (1993) [*Nat. Genet.*](javascript:AL_get(this, 'jour', 'Nat %0D%0AGenet.');) **3**, 252-255.
37. Gudmundsson, G., Hallgrímsson, J., Jónasson, T. A. and Bjarnason, O. (1972) *Brain* **95**, 387-404.
38. [Ghiso, J](http://www.ncbi.nlm.nih.gov/pubmed?term="Ghiso J"%5BAuthor%5D)., [Jensson, O](http://www.ncbi.nlm.nih.gov/pubmed?term="Jensson O"%5BAuthor%5D). and [Frangione, B](http://www.ncbi.nlm.nih.gov/pubmed?term="Frangione B"%5BAuthor%5D). (1986) *Proc. Natl. Acad. Sci. U S A* **83**, 2974-2978.
39. Ehrlich, J. C. and Ratner, I. M. (1961) *Am. J. Pathol.* **38**,49-59.
40. [Cooper, G. J](http://www.ncbi.nlm.nih.gov/pubmed?term="Cooper GJ"%5BAuthor%5D)., [Willis, A. C](http://www.ncbi.nlm.nih.gov/pubmed?term="Willis AC"%5BAuthor%5D)., [Clark, A](http://www.ncbi.nlm.nih.gov/pubmed?term="Clark A"%5BAuthor%5D)., [Turner, R. C](http://www.ncbi.nlm.nih.gov/pubmed?term="Turner RC"%5BAuthor%5D)., [Sim, R. B](http://www.ncbi.nlm.nih.gov/pubmed?term="Sim RB"%5BAuthor%5D). and [Reid, K. B](http://www.ncbi.nlm.nih.gov/pubmed?term="Reid KB"%5BAuthor%5D). (1987) *Proc. Natl. Acad. Sci. U S A* **84**, 8628-8632.
41. Hazard, J. B., Hawk, W. A. and Crile, G. Jr. (1959) [*J. Clin. Endocrinol. Metab.*](javascript:AL_get(this, 'jour', 'J Clin %0D%0AEndocrinol Metab.');) **19**, 152-161.
42. [Butler, M](http://www.ncbi.nlm.nih.gov/pubmed?term="Butler M"%5BAuthor%5D). and [Khan, S](http://www.ncbi.nlm.nih.gov/pubmed?term="Khan S"%5BAuthor%5D). (1986) [*Arch. Pathol. Lab. Med.*](javascript:AL_get(this, 'jour', 'Arch %0D%0APathol Lab Med.');) **110**, 647-649.
43. [Kaye, G. C](http://www.ncbi.nlm.nih.gov/pubmed?term="Kaye GC"%5BAuthor%5D)., [Butler, M. G](http://www.ncbi.nlm.nih.gov/pubmed?term="Butler MG"%5BAuthor%5D)., [d'Ardenne A. J](http://www.ncbi.nlm.nih.gov/pubmed?term="d'Ardenne AJ"%5BAuthor%5D)., [Edmondson S. J](http://www.ncbi.nlm.nih.gov/pubmed?term="Edmondson SJ"%5BAuthor%5D)., [Camm A. J](http://www.ncbi.nlm.nih.gov/pubmed?term="Camm AJ"%5BAuthor%5D). and [Slavin G](http://www.ncbi.nlm.nih.gov/pubmed?term="Slavin G"%5BAuthor%5D). (1986) [*Br. Heart J.*](javascript:AL_get(this, 'jour', 'Br Heart J.');) **56**, 317-320.
44. [Westermark, P](http://www.ncbi.nlm.nih.gov/pubmed?term="Westermark P"%5BAuthor%5D)., [Johansson, B](http://www.ncbi.nlm.nih.gov/pubmed?term="Johansson B"%5BAuthor%5D). and [Natvig, J. B](http://www.ncbi.nlm.nih.gov/pubmed?term="Natvig JB"%5BAuthor%5D). (1979) [*Scand. J. Immunol.*](javascript:AL_get(this, 'jour', 'Scand J Immunol.');) **10**, 303-308.
45. Bilbao, J. M., Horvath, E., Hudson, A. R. and Kovacs, K. (1975) *Arch. Pathol.* **99**, 411-415.
46. [Störkel, S](http://www.ncbi.nlm.nih.gov/pubmed?term="Störkel S"%5BAuthor%5D)., [Schneider, H. M](http://www.ncbi.nlm.nih.gov/pubmed?term="Schneider HM"%5BAuthor%5D)., [Müntefering, H](http://www.ncbi.nlm.nih.gov/pubmed?term="Müntefering H"%5BAuthor%5D). and [Kashiwagi, S](http://www.ncbi.nlm.nih.gov/pubmed?term="Kashiwagi S"%5BAuthor%5D). (1983) [*Lab. Invest.*](javascript:AL_get(this, 'jour', 'Lab %0D%0AInvest.');) **48**, 108-111.
47. Iwata, T., Kamei, T., Uchino, F., Mimaya, H., Yanagaki, T. and Etoh, H. [(1978)](http://www.ncbi.nlm.nih.gov/pubmed/676743) *Acta Pathol. Jpn.* **28**, 193-203.
48. Häggqvist, B., Näslund, J., Sletten, K., Westermark, G. T., Mucchiano, G., Tjernberg, L. O., Nordstedt, C., Engström, U. and Westermark, P. (1999) *Proc. Natl. Acad. Sci. U S A* **96**, 8669-8674.
49. [Tsutsumi, Y](http://www.ncbi.nlm.nih.gov/pubmed?term="Tsutsumi Y"%5BAuthor%5D)., [Serizawa, A](http://www.ncbi.nlm.nih.gov/pubmed?term="Serizawa A"%5BAuthor%5D). and [Hori, S](http://www.ncbi.nlm.nih.gov/pubmed?term="Hori S"%5BAuthor%5D). (1996) [*Pathol. Int.*](javascript:AL_get(this, 'jour', 'Pathol %0D%0AInt.');) **46**, 491-497.
50. [Klintworth, G. K](http://www.ncbi.nlm.nih.gov/pubmed?term="Klintworth GK"%5BAuthor%5D)., [Valnickova, Z](http://www.ncbi.nlm.nih.gov/pubmed?term="Valnickova Z"%5BAuthor%5D)., [Kielar, R. A](http://www.ncbi.nlm.nih.gov/pubmed?term="Kielar RA"%5BAuthor%5D)., [Baratz, K. H](http://www.ncbi.nlm.nih.gov/pubmed?term="Baratz KH"%5BAuthor%5D)., [Campbell, R. J](http://www.ncbi.nlm.nih.gov/pubmed?term="Campbell RJ"%5BAuthor%5D). and [Enghild, J. J](http://www.ncbi.nlm.nih.gov/pubmed?term="Enghild JJ"%5BAuthor%5D). (1997) [*Invest. Ophthalmol. Vis. Sci.*](javascript:AL_get(this, 'jour', 'Invest %0D%0AOphthalmol Vis Sci.');) **38**, 2756-2763.
51. [Ando, Y](http://www.ncbi.nlm.nih.gov/pubmed?term="Ando Y"%5BAuthor%5D)., [Nakamura, M](http://www.ncbi.nlm.nih.gov/pubmed?term="Nakamura M"%5BAuthor%5D)., [Kai, H](http://www.ncbi.nlm.nih.gov/pubmed?term="Kai H"%5BAuthor%5D)., [Katsuragi, S](http://www.ncbi.nlm.nih.gov/pubmed?term="Katsuragi S"%5BAuthor%5D)., [Terazaki, H](http://www.ncbi.nlm.nih.gov/pubmed?term="Terazaki H"%5BAuthor%5D)., [Nozawa, T](http://www.ncbi.nlm.nih.gov/pubmed?term="Nozawa T"%5BAuthor%5D)., [Okuda, T](http://www.ncbi.nlm.nih.gov/pubmed?term="Okuda T"%5BAuthor%5D)., [Misumi, S](http://www.ncbi.nlm.nih.gov/pubmed?term="Misumi S"%5BAuthor%5D)., [Matsunaga, N](http://www.ncbi.nlm.nih.gov/pubmed?term="Matsunaga N"%5BAuthor%5D)., [Hata, K](http://www.ncbi.nlm.nih.gov/pubmed?term="Hata K"%5BAuthor%5D)., [Tajiri, T](http://www.ncbi.nlm.nih.gov/pubmed?term="Tajiri T"%5BAuthor%5D)., [Shoji, S](http://www.ncbi.nlm.nih.gov/pubmed?term="Shoji S"%5BAuthor%5D)., [Yamashita, T](http://www.ncbi.nlm.nih.gov/pubmed?term="Yamashita T"%5BAuthor%5D)., [Haraoka, K](http://www.ncbi.nlm.nih.gov/pubmed?term="Haraoka K"%5BAuthor%5D)., [Obayashi, K](http://www.ncbi.nlm.nih.gov/pubmed?term="Obayashi K"%5BAuthor%5D)., [Matsumoto, K](http://www.ncbi.nlm.nih.gov/pubmed?term="Matsumoto K"%5BAuthor%5D)., [Ando, M](http://www.ncbi.nlm.nih.gov/pubmed?term="Ando M"%5BAuthor%5D). and [Uchino, M](http://www.ncbi.nlm.nih.gov/pubmed?term="Uchino M"%5BAuthor%5D). (2002) [*Lab. Invest.*](javascript:AL_get(this, 'jour', 'Lab %0D%0AInvest.');) **82**, 757-766.
52. [Solomon, A](http://www.ncbi.nlm.nih.gov/pubmed?term="Solomon A"%5BAuthor%5D)., [Murphy, C. L](http://www.ncbi.nlm.nih.gov/pubmed?term="Murphy CL"%5BAuthor%5D)., [Weaver, K](http://www.ncbi.nlm.nih.gov/pubmed?term="Weaver K"%5BAuthor%5D)., [Weiss, D. T](http://www.ncbi.nlm.nih.gov/pubmed?term="Weiss DT"%5BAuthor%5D)., [Hrncic, R](http://www.ncbi.nlm.nih.gov/pubmed?term="Hrncic R"%5BAuthor%5D)., [Eulitz, M](http://www.ncbi.nlm.nih.gov/pubmed?term="Eulitz M"%5BAuthor%5D)., [Donnell, R. L](http://www.ncbi.nlm.nih.gov/pubmed?term="Donnell RL"%5BAuthor%5D)., [Sletten, K](http://www.ncbi.nlm.nih.gov/pubmed?term="Sletten K"%5BAuthor%5D)., [Westermark, G](http://www.ncbi.nlm.nih.gov/pubmed?term="Westermark G"%5BAuthor%5D). and [Westermark, P](http://www.ncbi.nlm.nih.gov/pubmed?term="Westermark P"%5BAuthor%5D). (2003) [*J. Lab. Clin. Med.*](javascript:AL_get(this, 'jour', 'J Lab Clin%0D%0A Med.');) **142**, 348-455.
53. [Murphy, C. L](http://www.ncbi.nlm.nih.gov/pubmed?term="Murphy CL"%5BAuthor%5D&itool=EntrezSystem2.PEntrez.Pubmed.Pubmed_ResultsPanel.Pubmed_RVAbstract)., [Kestler, D. P](http://www.ncbi.nlm.nih.gov/pubmed?term="Kestler DP"%5BAuthor%5D&itool=EntrezSystem2.PEntrez.Pubmed.Pubmed_ResultsPanel.Pubmed_RVAbstract)., [Foster, J. S](http://www.ncbi.nlm.nih.gov/pubmed?term="Foster JS"%5BAuthor%5D&itool=EntrezSystem2.PEntrez.Pubmed.Pubmed_ResultsPanel.Pubmed_RVAbstract)., [Wang, S](http://www.ncbi.nlm.nih.gov/pubmed?term="Wang S"%5BAuthor%5D&itool=EntrezSystem2.PEntrez.Pubmed.Pubmed_ResultsPanel.Pubmed_RVAbstract)., [Macy, S. D](http://www.ncbi.nlm.nih.gov/pubmed?term="Macy SD"%5BAuthor%5D&itool=EntrezSystem2.PEntrez.Pubmed.Pubmed_ResultsPanel.Pubmed_RVAbstract)., [Kennel, S. J](http://www.ncbi.nlm.nih.gov/pubmed?term="Kennel SJ"%5BAuthor%5D&itool=EntrezSystem2.PEntrez.Pubmed.Pubmed_ResultsPanel.Pubmed_RVAbstract)., [Carlson, E. R](http://www.ncbi.nlm.nih.gov/pubmed?term="Carlson ER"%5BAuthor%5D&itool=EntrezSystem2.PEntrez.Pubmed.Pubmed_ResultsPanel.Pubmed_RVAbstract)., [Hudson, J](http://www.ncbi.nlm.nih.gov/pubmed?term="Hudson J"%5BAuthor%5D&itool=EntrezSystem2.PEntrez.Pubmed.Pubmed_ResultsPanel.Pubmed_RVAbstract)., [Weiss, D. T](http://www.ncbi.nlm.nih.gov/pubmed?term="Weiss DT"%5BAuthor%5D&itool=EntrezSystem2.PEntrez.Pubmed.Pubmed_ResultsPanel.Pubmed_RVAbstract). and [Solomon, A](http://www.ncbi.nlm.nih.gov/pubmed?term="Solomon A"%5BAuthor%5D&itool=EntrezSystem2.PEntrez.Pubmed.Pubmed_ResultsPanel.Pubmed_RVAbstract). (2008) *Amyloid* **15**, 89-95.
54. [Gustafsson, M](http://www.ncbi.nlm.nih.gov/pubmed?term="Gustafsson M"%5BAuthor%5D)., [Thyberg, J](http://www.ncbi.nlm.nih.gov/pubmed?term="Thyberg J"%5BAuthor%5D)., [Näslund, J](http://www.ncbi.nlm.nih.gov/pubmed?term="Näslund J"%5BAuthor%5D)., [Eliasson, E](http://www.ncbi.nlm.nih.gov/pubmed?term="Eliasson E"%5BAuthor%5D). and [Johansson, J](http://www.ncbi.nlm.nih.gov/pubmed?term="Johansson J"%5BAuthor%5D). (1999) [*FEBS Lett.*](javascript:AL_get(this, 'jour', 'FEBS %0D%0ALett.');) **464**,138-142.
