# Supplementary material for: Large Proteins Have a Great Tendency to Aggregate but a Low Propensity to Form Amyloid Fibrils
Source: PLoS One. 2011 Jan 13;6(1):e16075. doi: 10.1371/journal.pone.0016075 (PMC3020945; doi:10.1371/journal.pone.0016075)
Supplement: Table S2 — Database of peptides or proteins forming intracellular or extracellular non-amyloid deposits in human diseases. The names of the proteins, their sizes, their associated pathologies and references are reported. (DOC) [file pone.0016075.s002.doc]

| Table S2 – Peptides or proteins forming intracellular or extracellular non-amyloid deposits in human diseases ^a^ | | | | |
| --- | --- | --- | --- | --- |
| Full name of  peptide or protein | Short name of peptide or protein | Size  (number of residues) ^b^ | Diseases associated with the deposits  of the peptide or protein | Reference |
| Neurogenic locus notch homolog protein 3 ectodomain | Notch 3 ectodomain | 1589 | - Cerebral autosomal dominant arteriopathy with subcortical infarcts and leukoencephalopathy (CADASIL) | 1, 2 |
| fibronectin | FN | 2355 | - Fibronectin non-amyloid glomerulopathy | 3 |
| transactive response DNA-binding protein 43 | TDP-43 | 414 | - Frontotemporal lobar degeneration with ubiquitin-positive inclusions  - Amyotrophic lateral sclerosis | 4, 5 |
| Immunoglobulin type M | IgM | ~ 6750 ^c^ | - Multiple myeloma (Russell bodies) | 6, 7 |
| Immunoglobulin type G | IgG | ~ 1350 ^c^ | - Multiple myeloma (Russell bodies) | 6, 7 |
| Complement C1q subcomponent | C1q | 3996 ^c^ | - Lobular glomerulonephritis  - C1q nephropathy | 8 |
| Alanine:glyoxylate aminotransferase | AGT | 392 | - Primary hyperoxaluria type 1 | 9 |
| Tamm-Horsfall urinary glycoprotein (uromodulin) | THP | 590 | - Medullary cystic kidney disease - Familial juvenile hyperuricemic nephropathy - Glomerulocystic kidney disease | 10, 11 |
| Immunoglobulin gamma 1 heavy chain ^d^ | - | ~ 345 | - Heavy chain deposition disease | 12, 13 |
| **^a^ The peptides and proteins listed in the table include cases reported to form either extracellular or intracellular deposits with evidence for non-amyloid structure. Hemoglobin and serpins have been excluded from the analysis because they form fibrillar aggregates which are highly structured albeit non-amyloid.**  **^b^ Data refer to the processed polypeptide chains that deposit into aggregates, not to the precursor proteins.**  **^c^ Calculated considering all subunits forming the protein**  **^d^ This protein has also been reported to form amyloid deposits in heavy chain amyloidosis, but has been assigned to the group of non-amyloid-forming proteins due to the prevalence of the heavy-chain deposition disease.** | | | | |

**References**

1. [Baudrimont, M](http://www.ncbi.nlm.nih.gov/pubmed?term="Baudrimont M"%5BAuthor%5D)., [Dubas, F](http://www.ncbi.nlm.nih.gov/pubmed?term="Dubas F"%5BAuthor%5D)., [Joutel, A](http://www.ncbi.nlm.nih.gov/pubmed?term="Joutel A"%5BAuthor%5D)., [Tournier-Lasserve, E](http://www.ncbi.nlm.nih.gov/pubmed?term="Tournier-Lasserve E"%5BAuthor%5D). and [Bousser, M. G](http://www.ncbi.nlm.nih.gov/pubmed?term="Bousser MG"%5BAuthor%5D). (1993) [*Stroke*](javascript:AL_get(this, 'jour', %0D%0A'Stroke.');) **24**, 122-125.
2. Joutel, [A](http://www.ncbi.nlm.nih.gov/pubmed?term="Joutel A"%5BAuthor%5D&itool=EntrezSystem2.PEntrez.Pubmed.Pubmed_ResultsPanel.Pubmed_RVAbstract)., [Andreux, F](http://www.ncbi.nlm.nih.gov/pubmed?term="Andreux F"%5BAuthor%5D&itool=EntrezSystem2.PEntrez.Pubmed.Pubmed_ResultsPanel.Pubmed_RVAbstract)., [Gaulis, S](http://www.ncbi.nlm.nih.gov/pubmed?term="Gaulis S"%5BAuthor%5D&itool=EntrezSystem2.PEntrez.Pubmed.Pubmed_ResultsPanel.Pubmed_RVAbstract)., [Domenga, V](http://www.ncbi.nlm.nih.gov/pubmed?term="Domenga V"%5BAuthor%5D&itool=EntrezSystem2.PEntrez.Pubmed.Pubmed_ResultsPanel.Pubmed_RVAbstract)., [Cecillon, M](http://www.ncbi.nlm.nih.gov/pubmed?term="Cecillon M"%5BAuthor%5D&itool=EntrezSystem2.PEntrez.Pubmed.Pubmed_ResultsPanel.Pubmed_RVAbstract)., [Battail, N](http://www.ncbi.nlm.nih.gov/pubmed?term="Battail N"%5BAuthor%5D&itool=EntrezSystem2.PEntrez.Pubmed.Pubmed_ResultsPanel.Pubmed_RVAbstract)., [Piga, N](http://www.ncbi.nlm.nih.gov/pubmed?term="Piga N"%5BAuthor%5D&itool=EntrezSystem2.PEntrez.Pubmed.Pubmed_ResultsPanel.Pubmed_RVAbstract)., [Chapon, F](http://www.ncbi.nlm.nih.gov/pubmed?term="Chapon F"%5BAuthor%5D&itool=EntrezSystem2.PEntrez.Pubmed.Pubmed_ResultsPanel.Pubmed_RVAbstract)., [Godfrain, C](http://www.ncbi.nlm.nih.gov/pubmed?term="Godfrain C"%5BAuthor%5D&itool=EntrezSystem2.PEntrez.Pubmed.Pubmed_ResultsPanel.Pubmed_RVAbstract). and [Tournier-Lasserve, E](http://www.ncbi.nlm.nih.gov/pubmed?term="Tournier-Lasserve E"%5BAuthor%5D&itool=EntrezSystem2.PEntrez.Pubmed.Pubmed_ResultsPanel.Pubmed_RVAbstract). (2000) *J. Clin. Invest.* **105**, 597-605.
3. Yong, J. L., Killingsworth, M. C., Spicer, S. T. and Wu, X. J. (2009) Int. *J. Clin. Exp. Pathol.* **20**, 210-216.
4. Neumann, M., Sampathu, D. M., Kwong, L. K., Truax, A. C., Micsenyi, M. C., Chou, T. T., Bruce, J., Schuck, T., Grossman, M., Clark, C. M., McCluskey, L. F., Miller, B. L., Masliah, E., Mackenzie, I. R., Feldman, H., Feiden, W., Kretzschmar, H. A., Trojanowski, J. Q. and Lee, V. M. (2006) *Science* **314**, 130-133.
5. [Kerman, A](http://www.ncbi.nlm.nih.gov/pubmed?term="Kerman A"%5BAuthor%5D)., [Liu, H. N](http://www.ncbi.nlm.nih.gov/pubmed?term="Liu HN"%5BAuthor%5D)., [Croul, S](http://www.ncbi.nlm.nih.gov/pubmed?term="Croul S"%5BAuthor%5D)., [Bilbao, J](http://www.ncbi.nlm.nih.gov/pubmed?term="Bilbao J"%5BAuthor%5D)., [Rogaeva, E](http://www.ncbi.nlm.nih.gov/pubmed?term="Rogaeva E"%5BAuthor%5D)., [Zinman, L](http://www.ncbi.nlm.nih.gov/pubmed?term="Zinman L"%5BAuthor%5D)., [Robertson, J](http://www.ncbi.nlm.nih.gov/pubmed?term="Robertson J"%5BAuthor%5D). and [Chakrabartty, A](http://www.ncbi.nlm.nih.gov/pubmed?term="Chakrabartty A"%5BAuthor%5D). (2010) [*Acta Neuropathol.*](javascript:AL_get(this, 'jour', 'Acta %0D%0ANeuropathol.');) **119**, 335-344.
6. [Matthews, J. B](http://www.ncbi.nlm.nih.gov/pubmed?term="Matthews JB"%5BAuthor%5D). (1983) [*Br. J. Exp. Pathol.*](javascript:AL_get(this, 'jour', 'Br J Exp %0D%0APathol.');) **64**, 331-335.
7. [Shultz, L. D](http://www.ncbi.nlm.nih.gov/pubmed?term="Shultz LD"%5BAuthor%5D)., [Coman, D. R](http://www.ncbi.nlm.nih.gov/pubmed?term="Coman DR"%5BAuthor%5D)., [Lyons, B. L](http://www.ncbi.nlm.nih.gov/pubmed?term="Lyons BL"%5BAuthor%5D)., [Sidman, C. L](http://www.ncbi.nlm.nih.gov/pubmed?term="Sidman CL"%5BAuthor%5D)., [Taylor, S](http://www.ncbi.nlm.nih.gov/pubmed?term="Taylor S"%5BAuthor%5D). (1987) [*Am. J. Pathol.*](javascript:AL_get(this, 'jour', 'Am J %0D%0APathol.');) **127**, 38-50.
8. [Joh, K](http://www.ncbi.nlm.nih.gov/pubmed?term="Joh K"%5BAuthor%5D)., [Aizawa, S](http://www.ncbi.nlm.nih.gov/pubmed?term="Aizawa S"%5BAuthor%5D)., [Takahashi, T](http://www.ncbi.nlm.nih.gov/pubmed?term="Takahashi T"%5BAuthor%5D)., [Hatakeyama, M](http://www.ncbi.nlm.nih.gov/pubmed?term="Hatakeyama M"%5BAuthor%5D)., [Muto, S](http://www.ncbi.nlm.nih.gov/pubmed?term="Muto S"%5BAuthor%5D)., [Asano, Y](http://www.ncbi.nlm.nih.gov/pubmed?term="Asano Y"%5BAuthor%5D)., [Shimizu, H](http://www.ncbi.nlm.nih.gov/pubmed?term="Shimizu H"%5BAuthor%5D). and [Suzuki, R](http://www.ncbi.nlm.nih.gov/pubmed?term="Suzuki R"%5BAuthor%5D). (1990) [*Acta Pathol. Jpn.*](javascript:AL_get(this, 'jour', 'Acta %0D%0APathol Jpn.');) **40**, 913-921.
9. Danpure, C. J., Purdue, P. E., Fryer, P., Griffiths, S., Allsop, J., Lumb, M. J., Guttridge, K. M., Jennings, P. R., Scheinman, J. I., Mauer S. M. and Davidson, N. O. (1993) *Am. J. Hum. Genet.* **53**, 417-432.
10. [Rampoldi, L](http://www.ncbi.nlm.nih.gov/pubmed?term="Rampoldi L"%5BAuthor%5D)., [Caridi, G](http://www.ncbi.nlm.nih.gov/pubmed?term="Caridi G"%5BAuthor%5D)., [Santon, D](http://www.ncbi.nlm.nih.gov/pubmed?term="Santon D"%5BAuthor%5D)., [Boaretto, F](http://www.ncbi.nlm.nih.gov/pubmed?term="Boaretto F"%5BAuthor%5D)., [Bernascone, I](http://www.ncbi.nlm.nih.gov/pubmed?term="Bernascone I"%5BAuthor%5D)., [Lamorte, G](http://www.ncbi.nlm.nih.gov/pubmed?term="Lamorte G"%5BAuthor%5D)., [Tardanico, R](http://www.ncbi.nlm.nih.gov/pubmed?term="Tardanico R"%5BAuthor%5D)., [Dagnino, M](http://www.ncbi.nlm.nih.gov/pubmed?term="Dagnino M"%5BAuthor%5D)., [Colussi, G](http://www.ncbi.nlm.nih.gov/pubmed?term="Colussi G"%5BAuthor%5D)., [Scolari, F](http://www.ncbi.nlm.nih.gov/pubmed?term="Scolari F"%5BAuthor%5D)., [Ghiggeri, G. M](http://www.ncbi.nlm.nih.gov/pubmed?term="Ghiggeri GM"%5BAuthor%5D)., [Amoroso, A](http://www.ncbi.nlm.nih.gov/pubmed?term="Amoroso A"%5BAuthor%5D). and [Casari, G](http://www.ncbi.nlm.nih.gov/pubmed?term="Casari G"%5BAuthor%5D). (2003) [*Hum. Mol. Genet.*](javascript:AL_get(this, 'jour', 'Hum Mol %0D%0AGenet.');) **12**, 3369-3384.
11. [Bleyer, A. J](http://www.ncbi.nlm.nih.gov/pubmed?term="Bleyer AJ"%5BAuthor%5D&itool=EntrezSystem2.PEntrez.Pubmed.Pubmed_ResultsPanel.Pubmed_RVAbstract)., [Hart, T. C](http://www.ncbi.nlm.nih.gov/pubmed?term="Hart TC"%5BAuthor%5D&itool=EntrezSystem2.PEntrez.Pubmed.Pubmed_ResultsPanel.Pubmed_RVAbstract)., [Willingham, M. C](http://www.ncbi.nlm.nih.gov/pubmed?term="Willingham MC"%5BAuthor%5D&itool=EntrezSystem2.PEntrez.Pubmed.Pubmed_ResultsPanel.Pubmed_RVAbstract)., [Iskandar, S. S](http://www.ncbi.nlm.nih.gov/pubmed?term="Iskandar SS"%5BAuthor%5D&itool=EntrezSystem2.PEntrez.Pubmed.Pubmed_ResultsPanel.Pubmed_RVAbstract)., [Gorry, M. C](http://www.ncbi.nlm.nih.gov/pubmed?term="Gorry MC"%5BAuthor%5D&itool=EntrezSystem2.PEntrez.Pubmed.Pubmed_ResultsPanel.Pubmed_RVAbstract). and [Trachtman, H](http://www.ncbi.nlm.nih.gov/pubmed?term="Trachtman H"%5BAuthor%5D&itool=EntrezSystem2.PEntrez.Pubmed.Pubmed_ResultsPanel.Pubmed_RVAbstract). (2005) *Pediatr. Nephrol.* **20**, 824-827.
12. [Aucouturier, P](http://www.ncbi.nlm.nih.gov/pubmed?term="Aucouturier P"%5BAuthor%5D)., [Khamlichi, A. A](http://www.ncbi.nlm.nih.gov/pubmed?term="Khamlichi AA"%5BAuthor%5D)., [Touchard, G](http://www.ncbi.nlm.nih.gov/pubmed?term="Touchard G"%5BAuthor%5D)., [Justrabo, E](http://www.ncbi.nlm.nih.gov/pubmed?term="Justrabo E"%5BAuthor%5D)., [Cogne, M](http://www.ncbi.nlm.nih.gov/pubmed?term="Cogne M"%5BAuthor%5D)., [Chauffert, B](http://www.ncbi.nlm.nih.gov/pubmed?term="Chauffert B"%5BAuthor%5D)., [Martin, F](http://www.ncbi.nlm.nih.gov/pubmed?term="Martin F"%5BAuthor%5D). and [Preud'homme, J. L](http://www.ncbi.nlm.nih.gov/pubmed?term="Preud'homme JL"%5BAuthor%5D). (1993) [*N. Engl. J. Med.*](javascript:AL_get(this, 'jour', 'N Engl J %0D%0AMed.');) **329**, 1389-1393.
13. Khamlichi, A. A., Aucouturier, P., Preud'homme, J. L. and Cogné, M. (1995) *Eur. J. Biochem.* **229**, 54-60.
